# Multi-Source Perception, Intelligent Decision-Making, and Precision Control for Autonomous Agricultural Systems: A Comprehensive Review

**DOI:** 10.3390/s26175680

**Published:** 2026-09-07

**Authors:** Shida Zhang, Yong Zhu, Zhe Zhao, Jiawen Xu, Jiawei Zhang, Zhijian Zheng

**Affiliations:** 1 National Research Center of Pumps, Jiangsu University, Zhenjiang 212013, China; zhangshida@stmail.ujs.edu.cn (S.Z.); 2222511077@stmail.ujs.edu.cn (Z.Z.); 2222511095@stmail.ujs.edu.cn (J.X.); 2222511048@stmail.ujs.edu.cn (J.Z.); 2 Ningbo Fiber Inspection Institute, Ningbo Institute for Product and Food Quality Inspection, Ningbo 315000, China; a04130129@126.com

**Keywords:** autonomous agricultural systems, agricultural machinery, multi-source perception, intelligent decision-making, precision control

## Abstract

The rapid advancement of autonomous agricultural systems (AASs) is transforming modern agriculture, where labor shortages, sustainability imperatives, and demands for precision farming are driving the adoption of intelligent agricultural platforms. Agricultural production environments present uniquely challenging conditions for autonomous agricultural systems, including unstructured and dynamically changing terrain, biologically variable targets, unpredictable illumination and weather conditions, and safe human–machine coexistence. This review systematically investigates three cornerstone technologies: multi-source perception, intelligent decision-making, and precision control. Furthermore, typical agricultural operations, including soil tillage, planting, irrigation and drainage, fertilization, plant protection, harvesting, and agricultural product processing, are reviewed to illustrate their applications. Based on representative operational scenarios, the research progress and application characteristics of intelligent equipment in environmental perception, operational optimization, and control execution are summarized. Specifically, multi-source perception is evolving from isolated sensor-based acquisition toward multimodal and deep learning-enabled semantic scene understanding. Intelligent decision-making has evolved from experience-driven approaches toward physics-informed, data-driven, and knowledge-enhanced frameworks for adaptive operational optimization. Precision control has progressed from conventional PID control toward adaptive, learning-based, and digital twin-enabled control strategies, achieving robust high-precision closed-loop regulation. However, several critical challenges persist: limited cross-domain generalization and robustness of perception models under environmental distribution shift, constrained interpretability and trustworthiness of data-driven decision systems, and insufficient adaptability of control architectures under multi-disturbance coupled field conditions. To address these gaps, future research should prioritize multi-source heterogeneous data fusion and standardization, collaborative control frameworks integrating mechanistic knowledge with data-driven learning, and explainable artificial intelligence combined with agricultural domain expertise—advancing toward genuinely autonomous, trustworthy, and resilient agricultural systems.

## 1. Introduction

Autonomous agricultural systems (AASs), integrating intelligent agricultural machinery, autonomous ground vehicles, unmanned aerial vehicles (UAVs), robotic harvesters, and automated processing equipment, are transforming modern agriculture by enabling autonomous perception, decision-making, and operation in complex and dynamic farming environments [1]. Driven by increasing global food demand, labor shortages, and the urgent need for sustainable and high-precision agricultural production, AASs have emerged as a key technological pathway toward efficient, resilient, and intelligent farming [2]. Unlike structured industrial environments, agricultural production presents uniquely challenging conditions, including unstructured terrain, biologically variable targets, dynamically changing weather and illumination, and frequent human–machine interactions. These characteristics impose stringent requirements on environmental perception, intelligent decision-making, and adaptive control. As the physical foundation of AASs, agricultural machinery fundamentally influences production efficiency, resource utilization, and operational sustainability [3]. With the rapid integration of advanced sensing technologies, machine vision, artificial intelligence, and intelligent control, agricultural machinery is evolving from conventional mechanized equipment into autonomous systems capable of multi-source perception, intelligent decision-making, and precision control [4].

The development of intelligent agricultural machinery has undergone an evolutionary process from the isolated application of single-function modules to system-level integrated and coordinated operations [5]. Early research primarily focused on the isolated application of individual key technologies, such as sensor-based environmental perception, prescription map–based offline decision-making, and single-variable control methods employing proportional-integral-derivative (PID) controllers [6]. While these techniques can achieve local performance optimization in specific operational tasks, they still suffer from issues such as information isolation, delayed decision-making, and insufficient control coordination. Traditional image-processing approaches are appealing in their simplicity and in the transparency of their decision logic. A major limitation is the significant decline in robustness as the operating environment becomes increasingly complex and unpredictable [7,8]. Spectral analysis and multi-sensor fusion can significantly improve accuracy. But the cost of the required instrumentation is high, the data processing demands are considerable, and finding an acceptable balance between real-time throughput and engineering practicality has proven to be a persistent challenge [9]. Consequently, a single technological pathway is inadequate to meet the requirements for precise and stable operations in complex agricultural production environments.

With the deep integration of information technologies and intelligent algorithms, AASs have gradually evolved toward the coordinated development of multi-source information fusion, intelligent decision-making, and closed-loop control [10]. An intelligent technological framework centered on “perception–decision–control” has been established. In this framework, multi-source perception provides the foundation for real-time acquisition of crop, soil, environmental, and machinery-state information; intelligent decision-making drives the transition of operational parameters from experience-based settings to data-driven and model-driven optimization; and precision control ensures accurate execution of operations through dynamic regulation and closed-loop optimization. These key enabling technologies are applied throughout the entire process, including tillage, planting, irrigation and drainage, fertilization, crop protection, harvesting, and agricultural product processing [11]. Existing studies have shown that tillage depth detection is achieved by acquiring plow penetration depth and implement state information using angle, displacement, draft force, and attitude sensors. Adaptive regulation of tillage depth is then realized through PID control or model predictive control (MPC) [12,13]. Crop row detection and navigation are primarily conducted by extracting crop row centerlines using light detection and ranging (LiDAR), and deep learning (DL), thereby enabling autonomous operation of agricultural systems [14,15,16]. Precision irrigation is implemented by constructing evapotranspiration (ET) prediction models based on meteorological data, soil moisture information, and remote sensing data [17,18]. Crop nutrient inversion is commonly performed by estimating nitrogen content and growth indicators using hyperspectral data, UAV imagery, and machine learning (ML) [19]. Overall, intelligent agricultural machinery technologies have evolved from early single-point breakthroughs into complex system engineering characterized by multi-technology integration [20].

Given that the purposes of operation and agronomic demands differ for each, specific perception–decision–control structures have been established for all kinds of autonomous agricultural platforms. In plant protection systems, machine vision, spectral sensors and DL are used to identify crop, weed and pest-affected areas; spatially explicit application schemes are generated from prescription maps and real-time canopy sensing for variable-rate, target-selective spraying [21,22]. Vision, vibration, rotational speed, and loss sensors are used to monitor grain breakage rate, material loss, and blockage in harvesting systems. Drum speed, concave clearance and cleaning airflow are all regulated by fuzzy control or MPC [23,24]. Processing systems use image analysis, near-infrared spectroscopy and ML for quality inspection and grading recognition, as well as parameter optimization of drying and sorting [25,26,27,28]. The synergy between the general perception-decision-control architecture and domain-specific implementations continues to advance AASs toward greater precision, autonomy, and full-process automation.

The rest of this paper is organized as follows. Section 2 systematically analyzes multi-source perception, intelligent decision-making, and precision control as three core enabling technologies for AASs, focusing on their functional roles and methodological foundations. Section 3 surveys the application of these technologies across the sequence of agricultural operations: soil tillage, planting, irrigation and drainage, fertilization, plant protection, crop harvesting, and agricultural product processing. Section 4 identifies the chief obstacles to further progress and outlines directions for future research, organized around the cross-cutting challenges of perception robustness, decision trustworthiness, and control adaptability that define the frontier of autonomous agricultural systems.

## 2. Core Enabling Technologies for Autonomous Agricultural Systems

**Figure 1:**
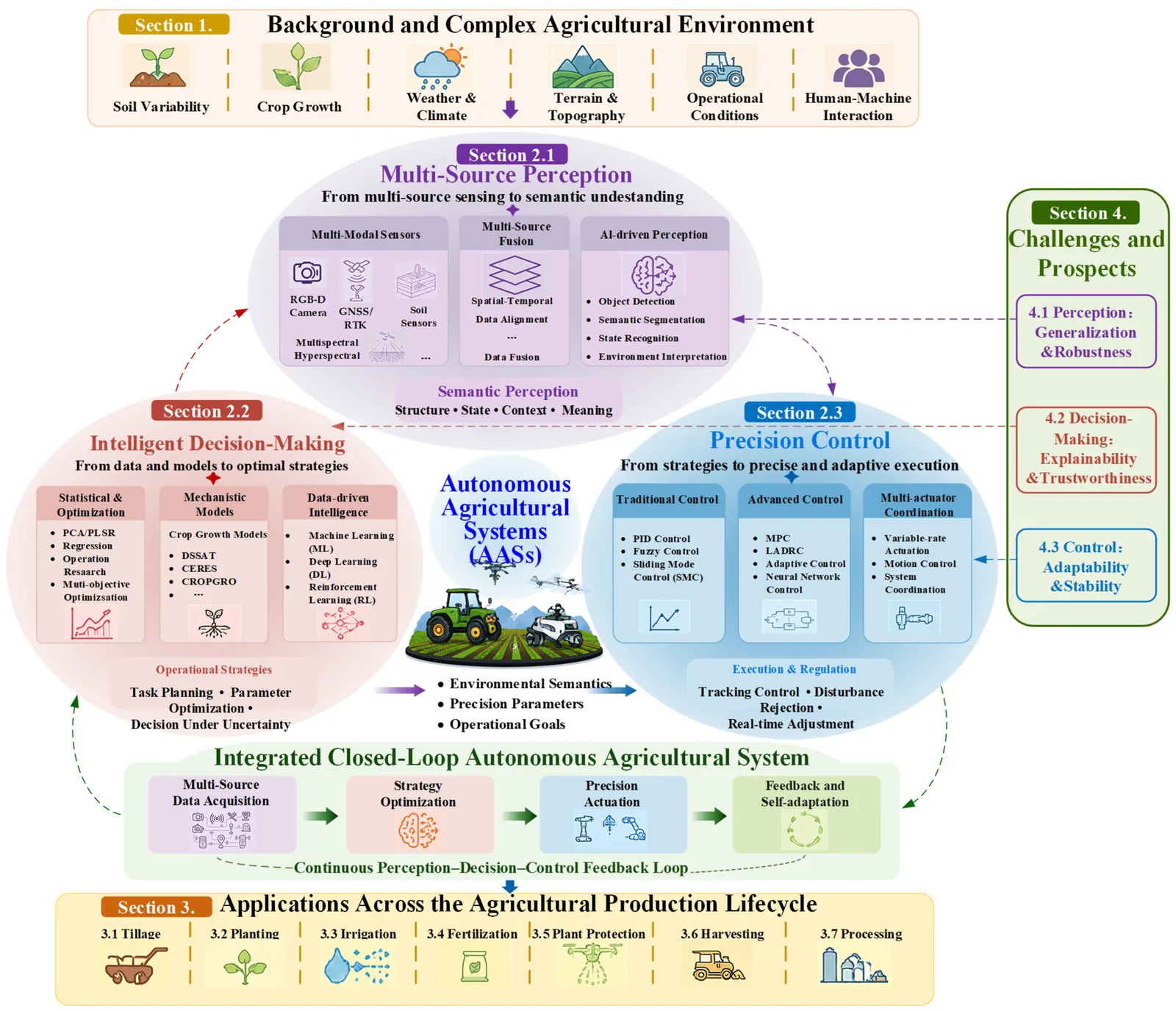
Hierarchical perception–decision–control collaborative architecture for AASs.

Unlike conventional automated agricultural machinery, AASs are characterized by the deep integration of perception, decision-making, and control capabilities. The perception module acquires multi-modal sensory information and generates semantically rich environmental representations. The decision-making module transforms these representations into adaptive operational strategies in response to environmental uncertainties. The control module implements these strategies through coordinated multi-actuator regulation and maintains system robustness via closed-loop feedback mechanisms. The core technical framework of AASs is thus structured around a three-tier collaborative architecture linking perception, decision, and control—each layer both enabling and constraining the performance of the others, as shown in Figure 1.

Rather than operating as independent modules, perception, decision-making, and control form a coupled closed-loop process [29]:

$$f_{p}(x_{k},\omega_{p})\to f_{d}({\hat{x}}_{k},\omega_{d})\to f_{c}({\hat{x}}_{k},u_{k},\omega_{c})\to x_{k+1}\to f_{p}(\cdot )$$

where *x_k_* denotes the system/environmental state, ${\hat{x}}_{k}$ its estimated representation, and *ω_p_*, *ω_d_*, and *ω_c_* represent perception uncertainty, decision uncertainty, and execution disturbances, respectively. This formulation highlights that the three functions are coupled not only through information and state feedback but also through the propagation of uncertainties and disturbances across the closed loop.

In the complex, dynamic, and uncertain agricultural environment, therefore, autonomous performance depends on the coordinated interaction of these three functions: sensing the environment intelligently, making informed operational decisions under uncertainty, and executing those decisions with high precision and adaptive robustness.

### 2.1. Multi-Modal Sensor Fusion and Environmental Perception

Accurate and robust perception of the operating environment constitutes the foundational layer of the AAS architecture: without reliable environmental perception, no downstream decision or control action can be trusted to produce the intended outcome. Multi-source perception technology, which provides this capacity, has followed a clear evolutionary path. It began with the use of single sensors to capture individual physical quantities, a configuration that could support only the most basic forms of automation. It then progressed to sensor fusion, in which data from multiple sources were combined to produce estimates more robust than any single sensor could provide. It is now entering a phase in which the goal is semantic perception: the direct transformation of multi-sensor data into operationally meaningful representations, increasingly through learned rather than hand-engineered transformations. Perception systems have gradually shifted toward multi-modal collaboration and high-level semantic feature representation [30]. By comprehensively sensing soil, crop, environmental, and equipment-status information, multi-source perception technology provides critical data support throughout the agricultural production lifecycle, covering tillage, sowing, irrigation and drainage, fertilization, plant protection, harvesting, and processing [31].

At the hardware layer, the perception architecture of AASs is built upon a multi-sensor framework that integrates machine vision, global navigation satellite systems (GNSS), LiDAR, multispectral/hyperspectral sensors, and inertial measurement units (IMUs), enabling robust and comprehensive perception of complex agricultural environments [32]. Consider the range of sensor types now available. Red, green, blue and depth (RGB-D) cameras and LiDAR supply geometric and structural information about the three-dimensional environment. Multispectral and hyperspectral imagers capture the physiological state of the crop through its spectral reflectance signature. Soil sensors measure moisture, compaction, and electrochemical properties. IMUs track the motion and orientation of the machine. Each sensor type provides a different window onto the agricultural environment, and each window is partially occluded under different conditions. Multiple sensing modalities are employed to achieve the above-mentioned effects of multi-modal perception. For example, in seeding and plant protection, camera and LiDAR data fusion can be used to identify a target and determine its spatial position in a single step, thereby improving the accuracy of variable-rate application [33]. For tillage and other soil-contacting work, a combination of mechanical sensor signals and IMU-derived pose information can be used to enhance real-time monitoring of working depth and load fluctuations [34]. Therefore, the primary goal of multi-source fusion is not simply to increase the number of data channels, but to exploit complementary information among heterogeneous sensors through appropriate fusion architectures. Depending on the application requirements, data-level, feature-level, and decision-level fusion strategies can be adopted to improve perception accuracy, robustness, and fault tolerance.

The way sensor data are turned into meaningful perceptual information is also changing significantly [35]. Traditional approaches based on image processing and spectral analysis rely on manually designed features and expert-defined rules. These methods offer advantages such as low computational requirements and interpretability, facilitating deployment on resource-constrained hardware [36]. The above characteristics are suitable for relatively well-structured or predictable environments in practice; thus, the feature set can be optimized according to the expected circumstances. But agriculture is not a controlled-environment system. In the messy, variable, and unpredictable conditions of real fields, hand-engineered features routinely fail to capture the diagnostic information they were designed for, and the result is a collapse in both generalization and robustness. This recognition has driven a paradigm shift toward DL, in which features are learned directly from data rather than specified in advance, thereby reshaping multi-source perception at the information-processing level [37]. In contrast, DL can extract high-level semantic features from complex backgrounds through end-to-end learning, demonstrating superior adaptability in tasks such as object detection, semantic segmentation, and state recognition [38]. Despite these advantages, DL remains highly dependent on large-scale annotated datasets, whereas the acquisition of high-quality data in agricultural scenarios is constrained by high costs. A comparative analysis of various multi-source perception technologies is illustrated in Table 1.

Multi-source perception systems generally integrate heterogeneous sensor information through three hierarchical fusion strategies: data-level fusion, feature-level fusion, and decision-level fusion. The selection of fusion architecture depends on sensor characteristics, information representation, and application requirements. Data-level fusion directly integrates raw measurements after calibration, spatial registration, and temporal synchronization, preserving maximum information but requiring accurate sensor alignment and high computational capability. These preprocessing procedures are essential because heterogeneous sensors usually operate with different sampling frequencies and coordinate systems, resulting in temporal and spatial inconsistencies during information integration [39]. Feature-level fusion extracts representative features from individual sensors and integrates them through mathematical models or learning-based networks. Compared with data-level fusion, feature-level fusion provides stronger capability for modeling nonlinear correlations among heterogeneous information and has become a dominant strategy in deep learning-based perception systems. For example, visual features from RGB cameras and geometric features from LiDAR can be jointly encoded to improve object recognition and localization accuracy [40]. However, deep learning-based feature fusion methods usually require sufficient labeled multi-source datasets, and limited data availability remains a major challenge for practical deployment in complex agricultural environments [41]. Decision-level fusion combines independent outputs from different sensors using methods such as weighted fusion, Bayesian inference, Dempster–Shafer (D-S) theory, and weighted voting [42]. These probabilistic and evidence-based approaches provide enhanced robustness against sensor uncertainty, individual sensor failures, and conflicting observations from heterogeneous sensors. A comparison of the three fusion strategies in terms of typical methods, advantages, and limitations is provided in Table 2.

### 2.2. AI-Driven Decision-Making and Operational Optimization Under Uncertainty

Intelligent decision-making serves as the critical cognitive bridge between perception and control in the AAS architecture. It is essentially defined by the transformation of multi-source information into operational strategies and control objectives. Unlike traditional static decision patterns based on experience or predefined rules, the decision-making systems of modern agricultural machinery are progressively evolving toward a dynamic optimization paradigm characterized by the integration of data-driven approaches and physical mechanisms [43]. Synergistic optimization of operational timing, trajectories, and key parameters can be achieved by fusing multi-source information, including soil, crop, environmental, meteorological, and equipment operational status. Consequently, foundational support is provided for precision agriculture and autonomous control.

Statistical and optimization methods are represented by principal component analysis (PCA), partial least squares regression (PLSR), and multiple regression. These methods are primarily utilized for multi-source data dimensionality reduction and critical variable extraction, and they play a significant role in revealing the correlations between environmental factors and crop growth. However, they are inadequate for characterizing complex nonlinear relationships [44]. Operational research seeks to optimize resource allocation and the way of operating through the construction of an objective function and constraints. A stable theoretical foundation for the multi-objective decision-making problem is provided by the above methods [45]. However, the performance of these methods is also constrained by the construction quality of the model, and their adaptability in complex, dynamic environments is relatively weak.

Mechanistic model-based approaches are typically represented by crop growth models (CGMs). By presenting the physiological and ecological processes of crops, environments and management practices, these models have clear physical meanings and are easily understood. Typical CGMs include the Decision Support System for Agrotechnology Transfer (DSSAT), Crop Estimation through Resource and Environment Synthesis (CERES) and Crop Growth Model for Legumes (CROPGRO), which have been widely used in yield prediction and optimization of water and fertilizer management [46]. However, these models require extensive parameterization, are difficult to tune, are sensitive to the environment, and thus do not meet the real-time requirements. Standalone mechanistic models provide limited real-time decision support for dynamic agricultural production.

Data-driven methods are represented by ML and reinforcement learning (RL). By constructing nonlinear mapping relationships between input variables and operational responses, these methods can effectively address uncertainty in complex systems and exhibit high precision in tasks such as yield prediction, path planning, and operational optimization [47]. RL is particularly suitable for sequential decision-making problems and enables strategy optimization in dynamic environments. However, the engineering application of these methods is constrained by a strong dependence on data quality and scale, as well as by insufficient model interpretability. A comparative analysis of the characteristics of various intelligent decision-making technologies is presented in Table 3.

### 2.3. Adaptive Precision Control and Multi-Actuator Coordination

Precision control serves as the execution core of AASs. Its primary objective is to achieve stable tracking and dynamic optimization of operational parameters under complex environmental conditions and disturbances. Higher requirements for control precision and real-time performance are imposed by intelligent agricultural systems, compared with traditional control systems [48,49]. Currently, control methods based on mechanistic modeling and data-driven approaches have been widely applied in critical operational stages. The consistency and refinement of operations have been effectively enhanced by these methods.

PID, fuzzy and sliding mode control (SMC) are still popular choices for the foundation of agricultural control systems due to their simple structure and low implementation cost among the control methodologies. These ways have been successfully used to control tillage depth and pose adjustment. However, the parameters are generally set by trial and error, and they perform poorly in highly nonlinear or time-varying systems [50]. With the development of control theory, MPC and neural network control methods have been introduced into AASs. Predictive control under multi-variable constraints is achieved by MPC through rolling optimization, making it suitable for complex coupled systems. Nonlinear dynamic characteristics can be approximated by neural network control to enable a degree of adaptive regulation [51]. However, these methods are challenged by high computational complexity and a strong dependence on models or data, and their widespread application in resource-constrained agricultural equipment remains a challenge. A systematic comparative analysis of various precision control technologies is presented in Table 4. High-frequency perception and low-latency response capabilities have been progressively acquired by precision control systems through the development of multi-sensor fusion and edge computing. Consequently, stable operation and improved overall reliability are maintained by these systems in complex field environments [52].

Nevertheless, the practical deployment of intelligent control methods requires consideration of the overall closed-loop latency rather than only algorithmic complexity. In autonomous systems, the response delay involves multiple components, including sensor acquisition, multi-source data fusion, model inference, communication, and actuator execution. Although learning-based methods provide strong nonlinear approximation and adaptive control capabilities, their inference latency may increase with model complexity and hardware limitations [53]. Similarly, MPC requires repeated online optimization within each control cycle, which may introduce considerable computational burden for systems with complex constraints or fast dynamics. Therefore, real-time implementation requires a trade-off among control accuracy, computational efficiency, and response speed. Lightweight neural networks, edge computing, model reduction, and fast optimization algorithms provide promising solutions for reducing end-to-end latency [54].

In addition to real-time implementation constraints, ensuring reliable closed-loop operation remains a fundamental challenge for learning-based control methods. Stability guarantees are difficult because data-driven policies may not preserve closed-loop stability under untrained conditions or distribution shifts. Therefore, Lyapunov-based verification and adaptive stability analysis are important for neural network controllers [55]. Moreover, robustness against disturbances and uncertainties, as well as explicit handling of actuator limitations and safety constraints, are essential for agricultural machinery operating in highly variable environments. Although MPC naturally handles constraints, its performance depends strongly on model accuracy and computational resources. Data-driven control methods further require safety assurance and convergence analysis due to exploration risks in physical systems [56]. Therefore, future autonomous agricultural systems should develop hybrid control frameworks that integrate physical models, learning mechanisms, and safety-guaranteed optimization.

## 3. Integrated Autonomous Systems Across the Agricultural Production Lifecycle

### 3.1. Soil Tillage Systems

Selecting appropriate tillage methods based on different crop types is important for improving soil structure, enhancing aeration and water retention, promoting root development, and increasing water use efficiency. Deep tillage and fine-soil preparation can increase the loosening of the soil, improve the soil structure, suppress weed growth, and create a good environment for high-yield and stable-production crops [57]. With the development of AASs, research on soil tillage systems is moving away from traditional experience-based methods and towards data-driven perception and intelligent control. Key enabling technologies include high-precision tillage depth sensing, autonomous tillage depth control, and soil resistance analysis.

#### 3.1.1. Tillage Depth Sensing and Adaptive Control

**Figure 2:**
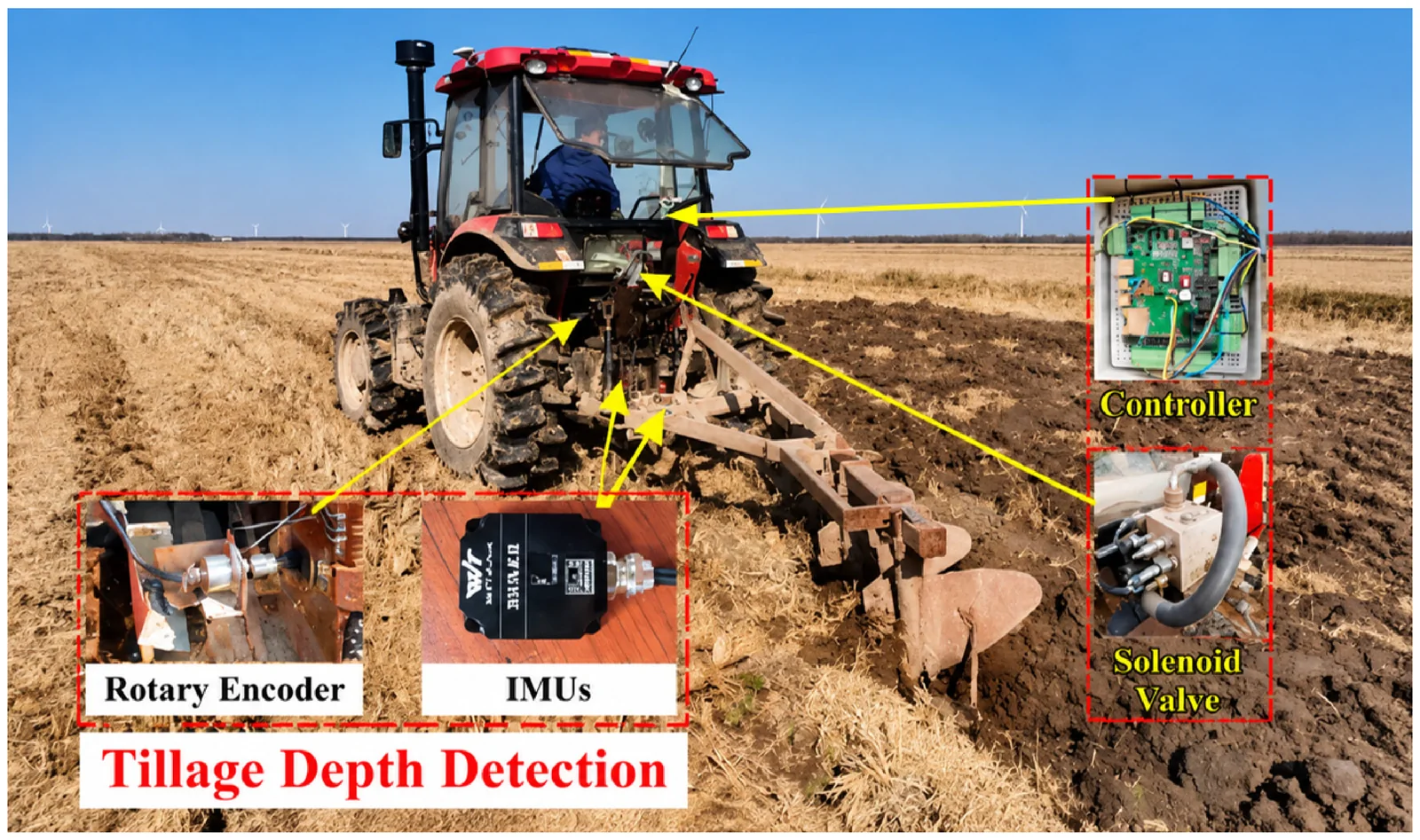
The autonomous soil tillage system [61].

Tillage depth not only affects soil inversion and fragmentation but also directly relates to the consistency of seeding depth and soil-seed contact, thus imposing critical constraints on seedling emergence quality and yield [58]. Related research is evolving from traditional single PID regulation to multi-parameter cooperative control, load sensing, and intelligent control. In 2023, Han et al. [59] designed a cooperative tillage depth control model. A fuzzy control strategy was introduced by this model to achieve closed-loop regulation between tillage depth and engine operating conditions, effectively suppressing overload phenomena. In 2023, Xiao et al. [60] developed a fuzzy PID tillage depth control system that integrated angular displacement and resistance sensing information, reducing tillage depth error and increasing soil fragmentation rate. In 2024, Zhu et al. [61] introduced an extended Kalman filter (EKF) for online parameter calibration in an attitude measurement detection model. Stable closed-loop regulation was achieved through recursive estimation and multi-sensor fusion to suppress vibration interference, combined with PID control. The autonomous control system is shown in Figure 2. In 2025, to improve system disturbance rejection and control accuracy, Tao et al. [62] designed an adaptive tillage depth control system for electric rotary tillers by integrating attitude and grating sensors with a LADRC strategy, achieving real-time closed-loop depth regulation and significantly improving tillage depth stability under variable field conditions. In 2025, Chen et al. [63] designed a visualized control platform. The platform integrated torque, vibration frequency, and inclination angle sensing, and B-PID control was employed to achieve digging depth trajectory tracking. In 2025, Guo et al. [64] proposed a self-excited/forced composite vibration subsoiler based on resistance feedback. Automatic switching of vibration modes was achieved by using tension sensing and adaptive threshold control, thereby reducing operating resistance and enhancing tillage depth stability.

#### 3.1.2. Soil–Tool Interaction Modeling and Parameter Optimization

In-depth research on soil–tool interaction mechanisms provides important theoretical support for structural optimization and parameter matching of autonomous soil tillage systems. Due to the spatial variability and nonlinear characteristics of soil, as well as the complex contact, fragmentation, and flow behaviors during soil-tool interaction, the mechanical response is difficult to describe accurately using traditional methods. Numerical simulation has become an important means to reveal tillage mechanisms [65]. Compared with analytical and semi-analytical methods, the discrete element method (DEM) can effectively characterize the discrete nature of soil particles and reveal soil disturbance mechanisms at the microscale. In 2022, to address the modeling of soil–tool interaction mechanisms and traction resistance prediction, Hoseinian et al. [66] developed a DEM-based model of a dual-side-wing subsoiling component. The Hertz-Mindlin and parallel bond models were introduced, and parameter calibration was completed in combination with soil bin tests, improving the prediction accuracy. In 2022, Kim et al. [67] established a full-scale tractor–plow–soil model, calibrating layered soil and tire parameters to predict traction resistance under different tillage depths with an accuracy of 90.8%. In 2023, Bae et al. [68] introduced the Edinburgh Elasto-Plastic Adhesion (EEPA) contact model to construct a tractor traction resistance prediction model that accounts for differences in soil physical and mechanical properties. The higher prediction accuracy and applicability of this model under various soil conditions were validated through field experiments. In 2024, using response surface methodology, Fang et al. [69] constructed an interaction model of a biomimetic elastic tooth, soil, and residual film, optimizing parameters such as tooth tip curvature, mounting angle, and spacing. In 2026, Kim et al. [70] considered measured soil bulk density, moisture content, and shear strength, calibrating the EEPA contact model with soil parameters. The variation patterns of traction resistance under different tillage depths were revealed.

Table 5 summarizes the progression from PID-based depth regulation to multi-sensor adaptive control and DEM-based soil–tool modeling, indicating a shift toward perception-driven autonomous tillage with enhanced robustness under variable field conditions.

### 3.2. Planting Systems

Autonomous planting systems must precisely regulate seed or seedling placement—position, depth, and spacing—under spatially and temporally varying soil conditions, terrain profiles, and operational speeds. The perception layer must extract navigation reference features (crop rows, ridge lines, furrows) from visual and ranging sensor data; the decision layer must translate perceived terrain and soil information into planting parameters; and the control layer must execute coordinated multi-actuator regulation to achieve the prescribed placement [71]. Consequently, operational quality and resource use efficiency are improved. Moreover, significant differences in seed morphology, frictional properties, and agronomic requirements are exhibited by different crops. Strong multi-parameter adaptive adjustment capabilities are required for precision seeding equipment.

#### 3.2.1. Navigation Perception and Reference Extraction

Navigation line recognition and operation target perception are the foundations for automatic operation of planting systems. DL and lightweight models have been widely used to identify crop rows, ridge lines, and tree trunks, providing navigation and positioning references for seeding, transplanting, and field management [72]. In 2024, Zhang et al. [73] proposed a lightweight convolutional neural network (CNN) model for seedling navigation. By improving feature extraction modules and incorporating a parameter-free attention mechanism, combined with the random sample consensus algorithm for navigation line fitting were enhanced. In 2024, Liu et al. [74] constructed a machine vision-based ridge line recognition method. This method extracted the region of interest, performed greyscale reconstruction, set a threshold and detect contours, and then used an approximate quadrilateral algorithm to extract ridge center lines for high-precision navigation recognition. In 2025, Lv et al. [75] introduced a lightweight improved model of YOLOv8. Optimization of the feature extraction structure and application of the least squares method for navigation line fitting reduced model complexity and improved recognition accuracy and real-time performance. In 2026, Zheng et al. [76] constructed a real-time detection and tracking model for tree trunks integrating YOLOv8 and ByteTrack. The positioning and triggering algorithm achieved dynamic positioning and control. The robustness and real-time performance of the model were enhanced by data augmentation and edge deployment, and thus, detection accuracy and trigger accuracy in complex environments were improved.

#### 3.2.2. Path Planning and Tracking Control

**Figure 3:**
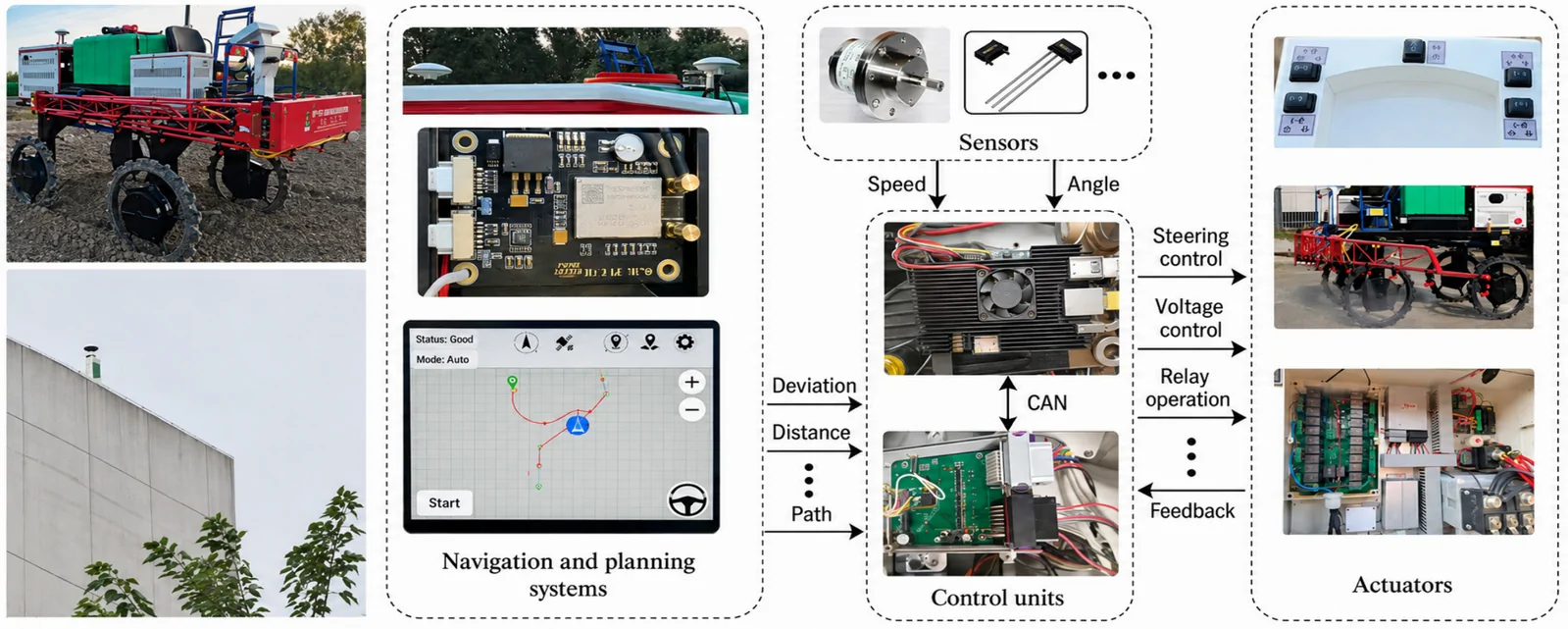
Autonomous navigation and path-tracking control system for agricultural vehicles [82].

Path tracking control stability is an important performance indicator. This technology enables stable operation of agricultural machinery along a predetermined trajectory by integrating navigation perception, vehicle kinematics modeling, and intelligent control algorithms. However, in complex field environments, factors such as soil slippage, speed fluctuations, and terrain undulations can weaken the robustness of traditional path tracking algorithms [77]. Commonly used path tracking methods include PID control, pure pursuit, Stanley algorithm, MPC, and SMC. In 2022, to address uncertain disturbances such as field sideslip for unmanned rice transplanters, Li et al. [78] constructed a path tracking control method incorporating a radial basis function (RBF) neural network, suppressing system chattering and improving lateral and heading tracking accuracy. In 2022, to address the difficulty of UAVs in anticipating small-scale obstacles, Ahmed et al. [79] proposed a data-driven dynamic obstacle avoidance method with multi-sensor fusion. A real-time path planning and state machine control framework was constructed to achieve adaptive obstacle avoidance and trajectory tracking. In 2023, Zheng et al. [80] designed a machine vision-based row-oriented spray system. By combining Hough transform and EKF for navigation line detection, and designing a delay compensation and closed-loop control strategy, the precise row-following spraying was achieved. In 2024, Liu et al. [81] proposed an ultrasonic sensing-based ridge-tracking method. In this method, abnormal ranging errors were suppressed by a clipping sliding window filter, and adaptive path tracking was realized by incorporating a fuzzy look-ahead distance decision. In 2025, Wang et al. [82] designed a self-adjusting look-ahead distance pure pursuit algorithm, optimizing the look-ahead distance through a kinematic model and error evaluation function. The autonomous navigation and path-tracking system is shown in Figure 3. In 2026, based on SMC, Song et al. [83] adopted extended line-of-sight guidance, using a state observer for speed estimation and sideslip compensation, enhancing system anti-disturbance capability and achieving high-precision stable tracking. In 2026, Cui et al. [84] introduced the sideslip angle of the center of mass into a kinematic model and constructed an MPC. The lateral error and sideslip oscillation were reduced under various path and velocity conditions, as validated through Carsim-Simulink co-simulation.

#### 3.2.3. Precision Seed/Seedling Placement and Multi-Actuator Coordination

Precision operation and variable control are key to improving the operational quality of autonomous planting systems. Technologies such as multi-sensor fusion, intelligent control, and digital twins are utilized to enable seeding and transplanting equipment to dynamically adjust seeding position, depth, and seedling picking/dropping actions. These adjustments are based on status information including soil conditions, operation trajectory, and implement attitude, achieving the precise placement of seeds or seedlings [85]. In 2024, Yao et al. [86] proposed a fully automatic transplanting cooperative operation method based on multi-sensor fusion and finite state machine. By coordinating the transverse conveying and longitudinal seedling picking mechanisms, they achieved precise tray positioning and continuous seedling picking/dropping, increasing the seedling placement success rate to 95%. In 2025, Shi et al. [87] developed a motion control system, integrating S-shaped acceleration/deceleration planning and fuzzy PID control to achieve smooth operation and high-precision positioning. In 2025, Li et al. [88] constructed a seeding depth detection and regulation system integrating multi-sensor information and fuzzy PID control. By fusing laser and array sensing information and performing online parameter adjustment, the seeding depth control accuracy and system stability were improved under complex terrain conditions. In 2025, Ahmad et al. [89] built a digital twin model of a rack-and-pinion vegetable seedling transmission device using virtual prototyping and co-simulation, introducing feedback control and parameter optimization to achieve precise trajectory tracking and position closed-loop control. In 2025, Yao et al. [90] developed a chassis height detection method based on ultrasonic ranging. Using echo characteristic analysis and error correction algorithms, the qualification rate of operation depth was increased to 94.4%.

#### 3.2.4. Mechatronic Coordination for Continuous Planting Operations

Currently, fully automatic transplanting systems still face challenges in engineering applications, including insufficient continuity of tray supply, low stability of seedling picking actuators, and limited coordination efficiency between picking and dropping [91,92]. Planting systems have been driven towards continuous operation, high efficiency, and low damage by the automation and mechatronic collaborative design. In 2021, Yang et al. [93] designed a mechatronic solution combining a belt-driven seedling conveying device and a programmable logic controller (PLC) control system. Precise positioning and stable operation of the conveying process were achieved through the collaborative optimization of motion mechanisms and control logic. In 2022, He et al. [94] constructed an automatic seedling feeding and transplanting cooperative control system based on a pre-treated seedling belt. Using an STM32 microcontroller for closed-loop speed regulation and actuator linkage control, the seedling miss rate was reduced. In 2024, Shi et al. [95] proposed a motor-cylinder coupled multi-row duckbill planting mechanism. Through kinematic modeling and simulation optimization of the end-effector structure, they facilitated coordinated matching between the drive system and operating components, reducing planting resistance while improving operational stability. In 2025, Chen et al. [96] designed a small electrically driven plug seedling transplanter, constructing an automated operation unit. System operational efficiency and stability were improved through the collaborative control of the motor and pneumatic systems. In 2026, Yao et al. [97] proposed a multi-layer tray handling and dual-claw cooperative picking. Based on trajectory planning and simulation analysis, combined with switchable acceleration/deceleration curves and a control system, continuous supply of seedling trays was ensured.

As summarized in Table 6, the convergence of lightweight DL architectures (CNN, YOLOv8) for crop row and ridge navigation line extraction with advanced path tracking strategies (adaptive SMC, kinematic MPC, fuzzy pure pursuit) and multi-sensor fusion-based seeding depth regulation constitutes the dominant technical pathway toward fully autonomous.

### 3.3. Drainage and Irrigation Systems

As the cost of water-resource acquisition rises, it will be difficult to provide uniform water distribution in autonomous irrigation systems and harm crop yields and resource-use efficiency. Therefore, improving the use efficiency of irrigation through good management of water resources has become a problem [98,99]. Precision irrigation technology is based on sensor monitoring, automatic control and information-based decision-making to regulate “when, where and how much” water is applied accurately, and its main technical components generally include soil moisture sensing, irrigation forecasting, water-demand decision-making and integrated water-and-fertilizer management systems [100,101].

#### 3.3.1. Water-Demand Prediction and Irrigation Decision-Making

The foundation of precision irrigation relies on accurate crop water demand prediction and intelligent irrigation decision-making enabled by multi-source data and advanced modeling approaches. Artificial intelligence models can be employed to extract underlying patterns from multi-source observational data. Therefore, technical support will be provided for crop water requirement estimation and irrigation scheme optimization in the absence of full mechanistic models or abundant meteorological data [102]. ET is the sum of water loss due to evaporation from the soil and transpiration by plants; it is frequently used to predict the water demand of crops and plan water-resource allocation. However, its prediction process is influenced by factors such as meteorological conditions, crop type, and soil status, exhibiting strong nonlinearity [103]. In 2024, to address the estimation of reference crop ET under insufficient meteorological data, Raza et al. [104] constructed a gene expression programming (GEP) model, designed 17 combinations of meteorological factors, and conducted comparative validation in different climatic zones of Pakistan. The results showed that the model has high prediction accuracy under multiple climatic conditions. In 2025, to address the strong nonlinearity and the data scarcity, Elbeltagi et al. [105] constructed a long-term actual ET prediction model by integrating a minimum redundancy maximum (MRMR) relevance feature selection method with a multi-structure neural network. The integration of decision support systems and ML is advancing the development of intelligent irrigation, enabling collaborative optimization of crop yield prediction and irrigation schemes. In 2025, to resolve the temporal constraints of canal water supply for cotton irrigation in arid regions, Wang et al. [106] employed the DSSAT framework incorporating the CERES and CROPGRO modules and coupled it with ML. To address the challenges of integrating dynamic weather, soil, and canal water supply information, a coupled model was developed to simulate cotton growth and optimize irrigation scheduling. In 2026, Hussain et al. [107] collected indoor experimental data on sprinkler irrigation uniformity coefficient and droplet diameter, and built multi-model prediction systems including random forest (RF), support vector regression (SVR), and multilayer perceptron (MLP) for fast prediction of irrigation uniformity and droplet characteristics.

#### 3.3.2. Closed-Loop Irrigation Control and Hydraulic Optimization

**Figure 4:**
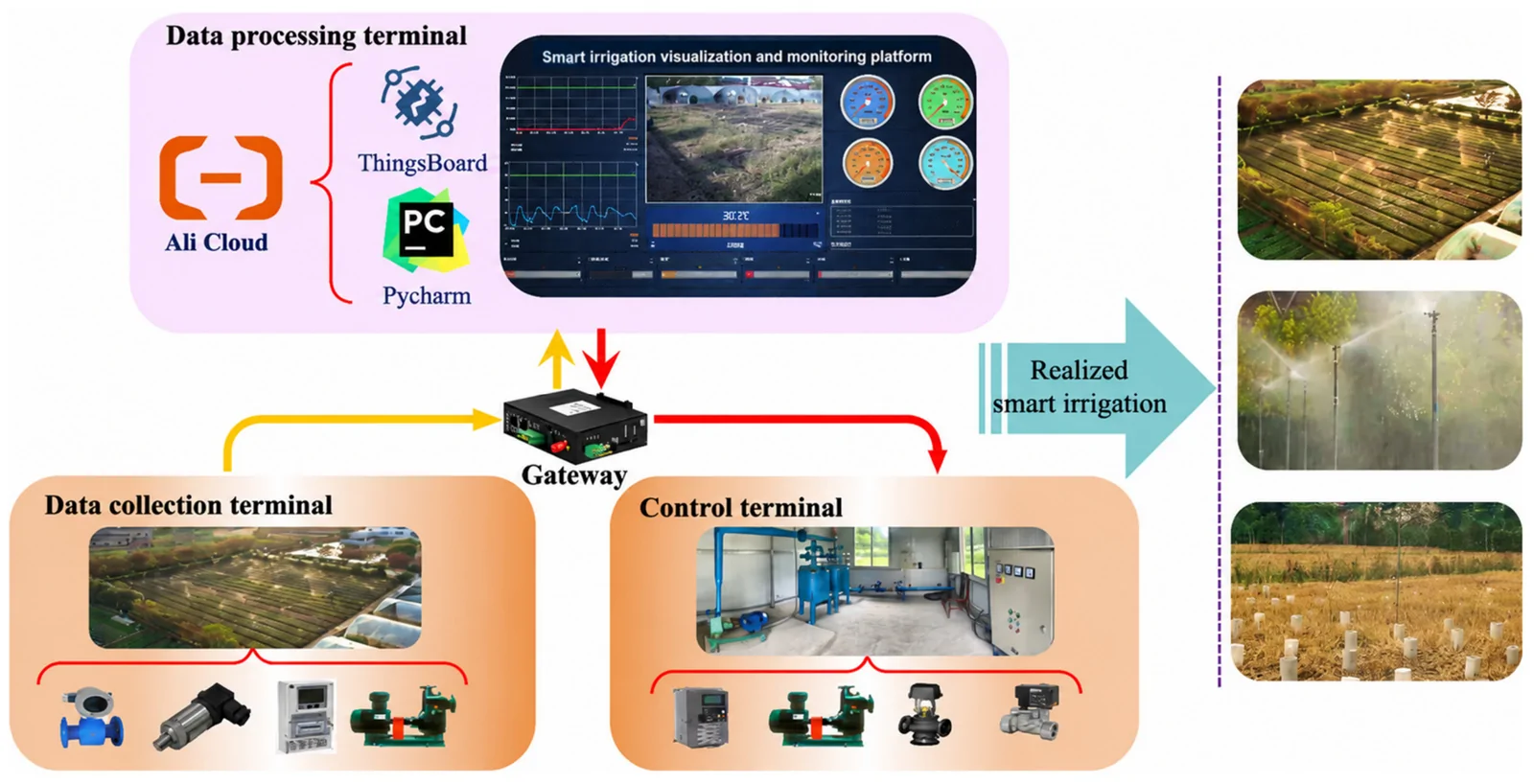
Composition of smart sprinkler irrigation system [109].

Precision irrigation systems are now transitioning from traditional open-loop control to closed-loop feedback and adaptive regulation. In 2018, Liu et al. [108] constructed a ZigBee wireless sensor network. The real-time substrate moisture detection module was integrated with a dynamic crop evapotranspiration model to trigger a threshold-based, multi-parameter coupled decision-making system for precise water-saving in soilless culture. In 2024, Pang et al. [109] set the sprinkler as the minimum optimization unit and used a genetic algorithm combined with variable-frequency pump control to optimize the rotational irrigation zones; thus, the pressure difference among sprinklers was effectively reduced, and 18% of the irrigation energy was saved, as shown in Figure 4. Structural optimization of key components and improvements in hydraulic performance have been investigated to enhance irrigation efficiency and uniformity [110]. In 2023, Pan et al. [111] designed a new type of automatic rotating sprinkler and added various water-flow-breaking devices. The spray characteristics were optimized through jet breakup and hydraulic performance tests, increasing sprinkler uniformity by 54.89%. In 2025, Xu et al. [112] built a water application superposition calculation model and conducted field validation. The nozzle diameter, travel speed, sprinkler rotation angle, and path spacing were optimized to increase the irrigation uniformity coefficient to 95.0%. In 2026, Tang et al. [113] improved the structure of the valve core through physical experiments, computational fluid dynamics (CFD) simulations, and the Box–Behnken response surface method to reduce the pressure drop by 22–33% and enhance the hydraulic performance of the system.

Table 7 shows the shift from empirical evapotranspiration models to ML prediction and sensor-based feedback control, enabling more efficient irrigation management.

### 3.4. Fertilization Systems

The rational allocation and efficient utilization of key nutrients such as nitrogen and potassium are central issues for achieving sustainable agricultural development. However, traditional fertilization methods generally suffer from excessive application, uneven distribution, and insufficient real-time control capability, leading to nutrient waste, soil degradation, and groundwater pollution [114]. Therefore, on-demand, quantitative, and uniform fertilization oriented toward the crop root zone has become an important direction for the development of intelligent fertilization systems. The key technologies mainly include crop nutrient sensing, precise control of fertilizer application rates, and variable-rate fertilization decision-making.

#### 3.4.1. Crop Nutrient and Fertilizer-Flow Perception

**Figure 5:**
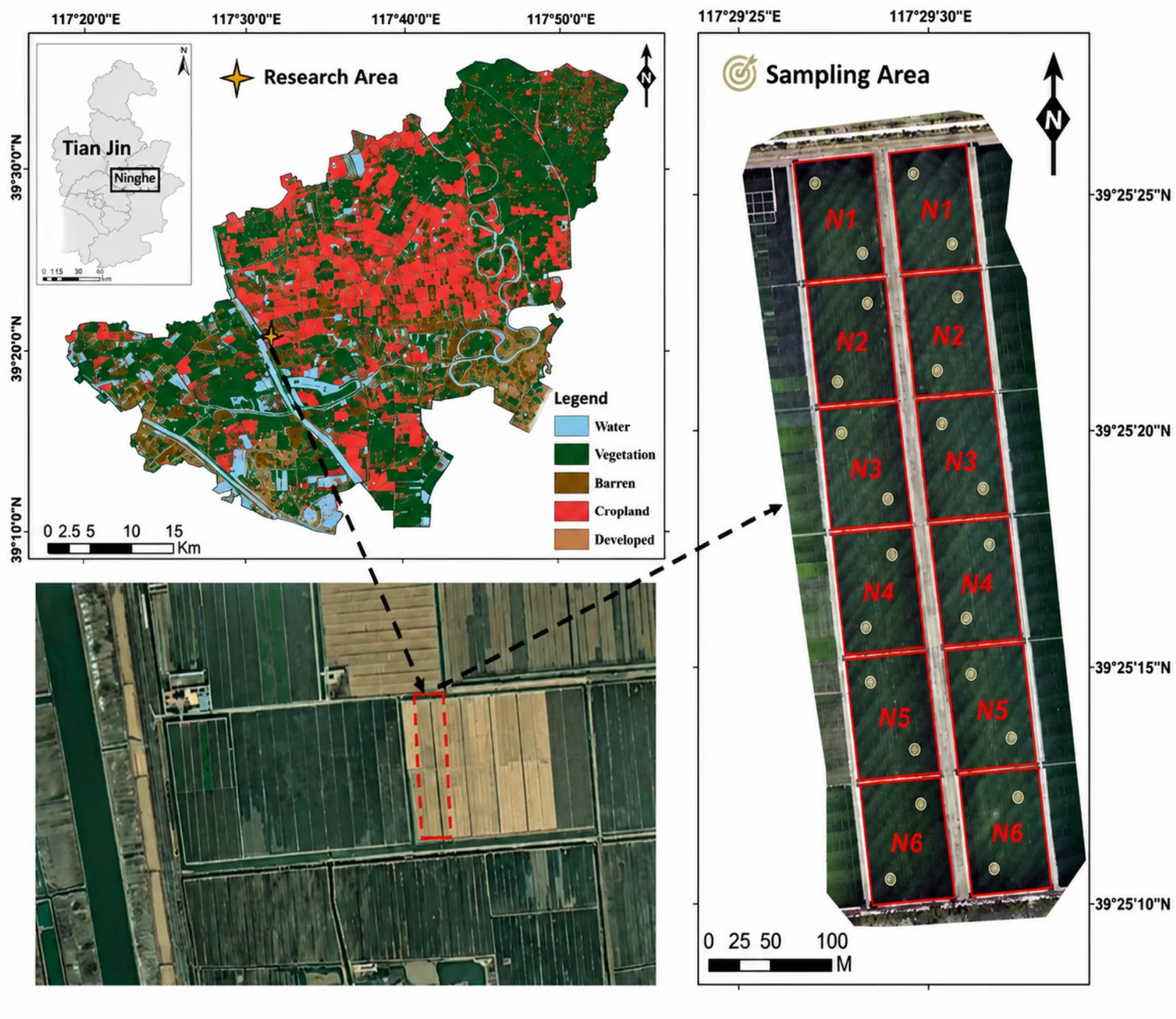
Geographical location and UAV sampling area layout [117].

In terms of crop nutrient sensing and fertilizer flow rate detection, relevant technologies are gradually advancing toward non-contact, high-precision, and multi-source fusion [115]. Leaf chlorophyll content is closely related to nitrogen nutrition status and can serve as an important indicator for decision-making in precision fertilization [116]. In 2023, Xu et al. [117] constructed a multi-dimensional feature fusion method based on UAV multi-source imagery, as shown in Figure 5. Grey relational analysis was used to screen key variables, and a parallel multi-branch deep neural network (DNN-F2) was constructed to explore the nonlinear synergistic relationships among features, achieving high-precision inversion and prediction. In 2023, Elsayed et al. [118] constructed multi-source spectral indices based on hyperspectral reflectance data. By utilizing a gradient boosting regression model, precise inversion of chlorophyll, yield, and quality parameters for sugar beet under varying nitrogen levels was achieved. The regulatory mechanism of nitrogen on crop physiology and spectral response was revealed. In 2023, to overcome the insufficient accuracy in identifying outliers in large-scale soil nitrogen data, Zheng et al. [119] developed a hybrid detection framework integrating isolation forest and local spatial autocorrelation analysis, enabling collaborative identification of global and local anomalies. The accuracy and reliability of anomaly detection were enhanced in the soil data experiment. In 2024, using UAV multispectral imagery and combining with support vector machine (SVM) and RF, Zhang et al. [120] constructed a chlorophyll inversion method. Rapid monitoring of crop nitrogen status was facilitated across different varieties and nitrogen stress conditions. In 2025, to resolve the challenge of real-time particle flow rate detection in centrifugal fertilization, Zhu et al. [121] proposed an improved YOLOv5s-seg visual detection method, achieving accurate particle segmentation and flow calculation through lightweight network and time-series imaging. In 2025, through the fusion of leaf and canopy-scale spectral data, Sun et al. [122] constructed a multi-feature ML inversion model. The inversion accuracy of orchard canopy nitrogen content was enhanced by performing multi-scale feature transformation via the 4SAIL model and CIELAB color space.

#### 3.4.2. Variable-Rate Fertilization Decision-Making and Adaptive Control

In terms of fertilizer application rate control and variable-rate fertilization, the uniformity and control accuracy remain challenging due to the complex flow characteristics of fertilizer granules and the pronounced pulsation during the discharge process [5]. In 2022, to address the problems of over-application and uneven fertilizer distribution in maize fertilizing seeders, Wang et al. [123] constructed an online fertilization rate detection model based on capacitive sensing principles. Through the integration of the relationship between fertilizer flow and capacitance response with a PID closed-loop control strategy, real-time sensing and dynamic adjustment of the fertilization process were achieved. In 2024, based on piecewise linear interpolation, Gao et al. [124] designed an adaptive fertilization control strategy. A dynamic mapping model between the motor drive signal and the fertilizer discharge shaft speed was established to realize adaptive adjustment and multi-parameter collaborative control. In 2025, Yu et al. [125] constructed a crop nutrient regulation framework based on ecological stoichiometry theory, coupled the DSSAT crop model with the XGBoost algorithm to achieve collaborative optimization control of nitrogen and phosphorus ratios. In 2025, Yu et al. [126] proposed a staged nitrogen optimization strategy through the coupling of the DSSAT model with a genetic algorithm.

Table 8 demonstrates a paradigm shift from uniform, experience-based fertilizer application toward sensor-driven, model-optimized variable-rate nutrient management, with the coupling of crop growth models (DSSAT) and ML algorithms (XGBoost, genetic algorithms) emerging as a key enabler for real-time, site-specific nitrogen-phosphorus ratio optimization and nutrient use efficiency improvement.

### 3.5. Plant Protection Systems

With the development of sensor technology and artificial intelligence, Autonomous plant protection systems are transitioning from traditional uniform spraying toward intelligent and precision directions. Intelligent plant protection equipment integrates multi-source sensing information and data analysis algorithms to achieve real-time perception of crop growth status, diseases, pests, weeds, and environmental parameters. By integrating image recognition and automated control technologies, the targeted pesticide application and the regulation of spray parameters are realized. Consequently, the quantity of pesticides used is reduced, and both their effectiveness and convenience in operation have been enhanced [127]. At present, most research on the intelligence of plant protection systems focuses on field information sensing, identification of pests, diseases and weeds, and implementation of precision spraying and variable-rate application.

#### 3.5.1. Crop and Target Perception for Plant Protection

Field perception is the foundation of autonomous crop protection systems. Machine vision, infrared imaging and ultrasonic sensors can be used to acquire data on canopy coverage, weed distribution, and areas affected by diseases and pests to provide target identification and spray rate adjustment support [128]. In 2022, by utilizing vegetation spectral reflectance and fluorescence characteristics, Wang et al. [129] used the spectral reflectance and fluorescence characteristics of vegetation to develop a low-cost vegetation sensor. By optimizing light source and band parameters based on comparisons between multi-band and single-band detection methods, the sensor achieved 100% detection accuracy under both dark and outdoor conditions. In 2024, Chen et al. [130] developed an automatic threshold segmentation method to build a weed identification algorithm for cotton fields and effectively differentiate between cotton seedlings and weeds. In 2025, Ou et al. [131] proposed a canopy leaf area density detection method that integrates LiDAR point-cloud-based leaf fitting and modeling with coupled multifactor regression. Preprocessing of point clouds, clustering and segmentation, and geometric reconstruction were used to achieve high-precision inversion of orchard canopy structure parameters. In 2025, an automatic liquid replenishment and docking station for field sprayers was designed by Gao et al. [132]. The system used visual servoing and an improved version of the YOLOv5 detection algorithm. Fill-port identification, robotic arm docking, and the liquid transfer itself were handled without human involvement.

#### 3.5.2. Precision Spraying and Variable-Rate Control

**Figure 6:**
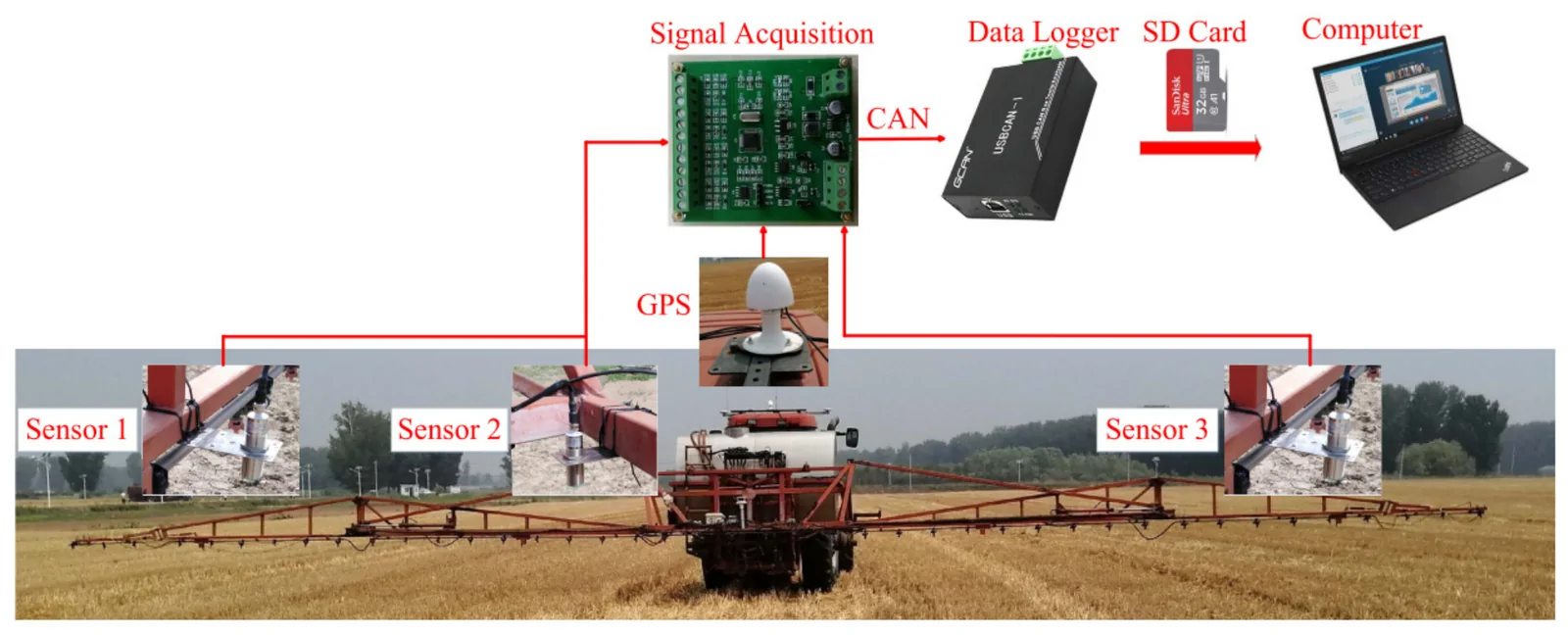
Sprayer boom height detection system architecture [136].

Precision spraying and variable-rate application technologies strongly influence both pesticide use efficiency and deposition uniformity. By bringing operational parameters into alignment with the spatiotemporal pattern of droplet deposition, researchers have been able to deliver chemicals on demand, zone by zone, and target by target, with measurable gains in uniformity [133]. In 2021, Liu et al. [134] tackled the problem of time-varying disturbances and model uncertainty in rate control by building a variable-rate system around a CNN that performs weed identification in real time and triggers targeted spraying. The architecture was realized through the integration of image acquisition with the actuation hardware, leading to greater spraying precision and reduced chemical consumption. In 2021, a precision control strategy employing LADRC was put forward by Ji et al. [135]. An extended-state observer was employed for online disturbance estimation and compensation, while particle swarm optimization (PSO)-based tuning of controller parameters achieved faster dynamic response and improved steady-state accuracy. In 2021, Dou et al. [136] developed a method for the dynamic detection and control of spray boom height based on ultrasonic sensing and data-driven signal processing. The system acquired operating conditions and boom-angle data in real time, applied an optimized signal-conditioning scheme, and thereby maintained stable boom height regulation. The spray boom height detection system is shown in Figure 6. In 2023, Li et al. [137] developed a breakpoint continuous spraying method that combined satellite navigation augmentation technology, wheel encoder-based positioning, lag compensation models and PID flow closed-loop control; this method was able to reduce both overlap and omission in spray coverage.

The studies collected in Table 9 reveal that the convergence of deep learning-based weed and disease identification (CNN, YOLOv5), LADRC for spray pressure regulation, and GNSS-guided breakpoint continuous spraying forms the technical foundation for achieving target-selective, dose-optimized agrochemical application with reduced environmental impact in autonomous crop protection.

### 3.6. Crop Harvesting Systems

Autonomous harvesting systems represent a type of agricultural equipment with complex structures, high operational loads, and prominent demands for intelligence. Taking combine harvesters as an example, they often operate under harsh conditions characterized by dust, vibration, and variable loads. Issues such as the loosening of critical connectors, clogging, and the degradation of cleaning performance frequently arise. These problems not only affect operational reliability and harvesting efficiency but may also cause downtime losses during critical agricultural periods [138]. Therefore, state perception, fault detection and diagnosis, and precise control of operational parameters for crop harvesting machinery have become important directions.

#### 3.6.1. Crop and Harvesting-Quality Perception

In terms of crop object perception and online harvesting quality detection, technologies such as machine vision, RGB-D cameras, LiDAR, ultrasonic sensors, and UAV remote sensing are widely used to perceive harvesting performance parameters including crop height, cutting width, lodged areas, and grain impurity rate [139]. In 2023, based on an improved YOLOX network and RGB-D fusion strategy, Hu et al. [140] developed a visual detection and three-dimensional localization method for fruit-picking robots. A synergistic enhancement of both target recognition accuracy and real-time performance was achieved in complex orchard environments. With the development of DL and edge computing platforms, relevant methods are transitioning from offline recognition to real-time embedded deployment. In 2024, based on an RGB-D camera, YOLOv5 instance segmentation, and the Jetson Xavier NX edge computing platform, Cui et al. [141] constructed an automatic grain unloading system for tracked rice combine harvesters, enabling the recognition of the unloading area and the mapping control of grain pile height and unloading time. In 2024, based on vehicle-mounted binocular vision and an improved YOLOv5s algorithm, Zhang et al. [142] developed an integrated system for wheat ear recognition, stubble height estimation, and rapid feed rate prediction. Through the integration of an efficient multi-scale attention mechanism, the C3Res2NetBlock multi-scale feature extraction module, and a P2 small-target detection layer, the performance of the system was optimized. In 2026, Liu et al. [143] constructed a lightweight semantic segmentation network StarNet-RiceSeg, which integrates high-dimensional feature mapping through Star Operation and a residual spatial attention mechanism. The network was deployed on the Jetson Xavier NX platform using TensorRT, achieving real-time and accurate identification of rice lodging areas. In 2026, Cui et al. [144] established a multimodal feature fusion model, ImpurityWeightNet. By integrating multi-scale image features with structural attributes such as area, aspect ratio, and perimeter, the high-precision estimation of impurity mass in the grain flow of combine harvesters was realized.

#### 3.6.2. Machine-State Monitoring and Fault Diagnosis

In fault detection and diagnosis, data-driven methods have become an important approach for condition monitoring. Operational data is collected using multi-source sensors, such as vibration, acoustic, strain, rotational speed, and image sensors. Subsequently, fault pattern recognition and condition diagnosis are achieved by combining signal preprocessing, time/frequency/time–frequency domain feature extraction, dimensionality reduction analysis, and ML. In 2022, Ma et al. [145] fused multi-point vibration signals with time–frequency domain feature extraction methods. By combining PCA with extreme learning machine (ELM), RF, and SVM classification models, rapid identification of threshing load status and clogging warning for combine harvesters were facilitated. In 2025, Lian et al. [146] proposed a method based on multi-point triaxial vibration signal acquisition and time–frequency feature analysis. Utilizing the integration of a wireless acquisition system and a Bayesian-optimized SVM model, precise identification of the operational status of conveyor trough bolts was achieved. In 2025, Ma et al. [147] constructed a multi-sensor acquisition system, fusing bearing vibration and rotational speed signals. The system employed wavelet decomposition, threshold discrimination, and an SVM model to achieve precise monitoring of clogging conditions in a potato-soil separation device.

#### 3.6.3. Adaptive Control of Harvesting Parameters

**Figure 7:**
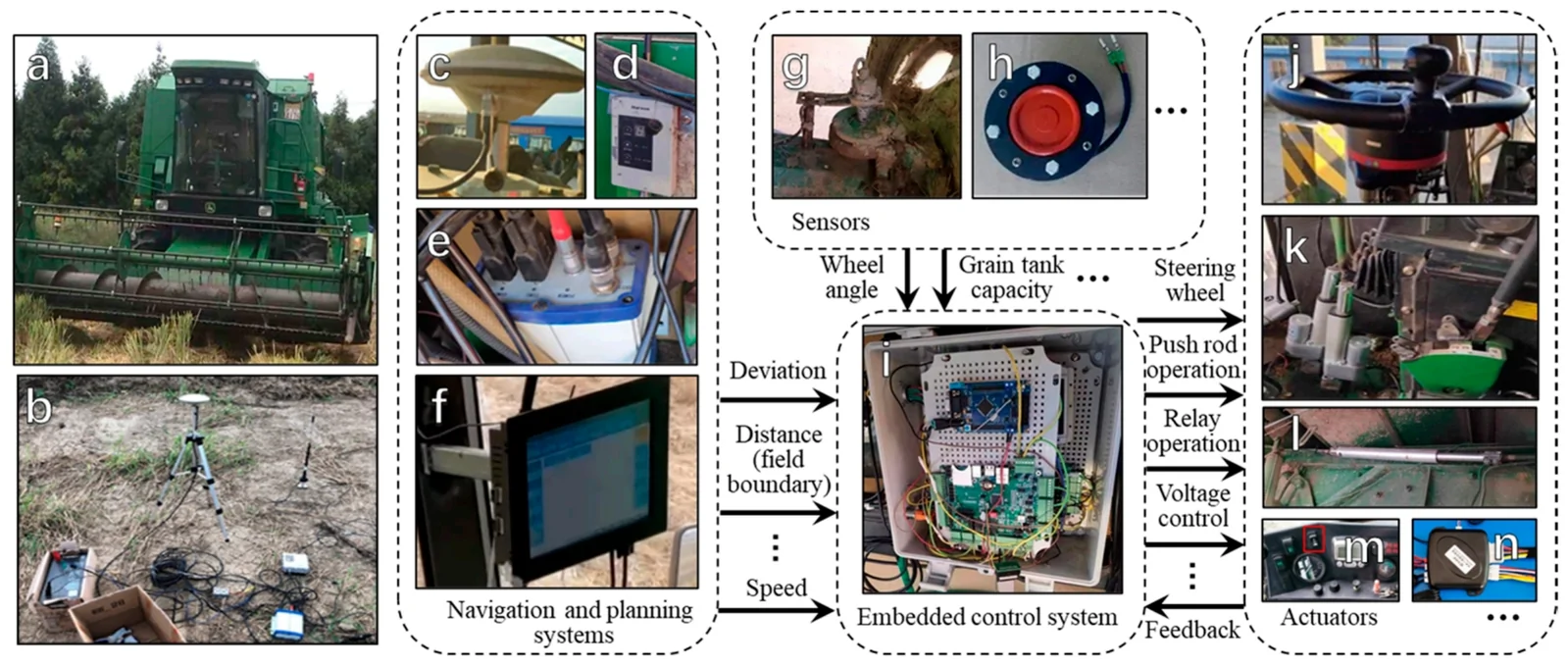
Composition of the automatic navigation system of the combine harvester [152]. (**a**)—Autonomous driving harvester; (**b**)—RTK base station; (**c**)—GPS antenna; (**d**)—RTK radio; (**e**)—GPS-RTK decoding box; (**f**)—Industrial control computer; (**g**)—Wheel angle sensor; (**h**)—Grain tank sensor; (**i**)—Embedded control cabinet; (**j**)—Electric steering wheel; (**k**)—Header lift push rod (left) and infinitely variable speed push rod (right); (**l**)—Threshing system clutch push rod; (**m**)—Electronic throttle; (**n**)—Remote start/stop relay module.

Traditional combine harvesters rely on mechanical transmission systems for power distribution. However, the limited speed regulation range, dynamic response, and overload protection capacity make it difficult for these systems to adapt to rapid changes in feed rate, moisture content, lodging degree, and terrain conditions [148]. Intelligent control systems have been able to achieve adaptive adjustment of key operational parameters such as header height and forward speed. In 2025, Zhang et al. [149] constructed a speed precise control system for unmanned combine harvesters based on multi-component slip rate monitoring, SVM fault prediction, and fuzzy logic speed control. In 2025, Qian et al. [150] developed a dynamic and electric drive model for a permanent magnet synchronous motor-driven threshing drum, adopting recursive least squares inertia identification and PI parameter self-tuning strategies to achieve adaptive and stable control of drum speed. In 2025, Liu et al. [151] constructed a precise control system for a hydraulic variable-diameter threshing drum. Based on real-time monitoring of oil pressure and threshing clearance, an adaptive variable-universe fuzzy PID controller was designed to achieve online adjustment of threshing clearance under varying feed rates. In 2025, Zhang et al. [152] proposed an driving system for a large-width rear-wheel steering combine harvester. The proposed system integrated RTK-GPS, feed-forward PID steering control, and a forward-looking Ackermann path-tracking algorithm to achieve high-precision tracking for both AB lines and circular operation paths. The integrated autonomous navigation system of the combine harvester is illustrated in Figure 7. In 2025, based on GPS, IMU, and EKF multi-sensor speed measurement, Chen et al. [153] proposed a long short-term memory (LSTM) network-based operational speed prediction model. By incorporating incremental PID closed-loop control, adaptive adjustment of operating speed and efficiency optimization were achieved.

Table 10 summarizes the transition toward multi-modal sensing, learning-based fault diagnosis, and adaptive control for autonomous harvesting under variable field conditions.

### 3.7. Agricultural Processing Systems

Agricultural processing systems underpin the shift from farm production to food manufacturing. The degree of automation directly affects processing efficiency, product uniformity, and other performance indicators. Typical processing operations include cleaning, grading, shelling, dehulling, drying, storage, and grinding. The above processing operations have the following features: strong process continuity, wide material variation, and high-quality requirements [154]. Consequently, process monitoring, online quality inspection, performance prediction, and intelligent control have been integrated into a unified framework.

#### 3.7.1. Multi-Source Process Monitoring and Online Quality Inspection

**Figure 8:**
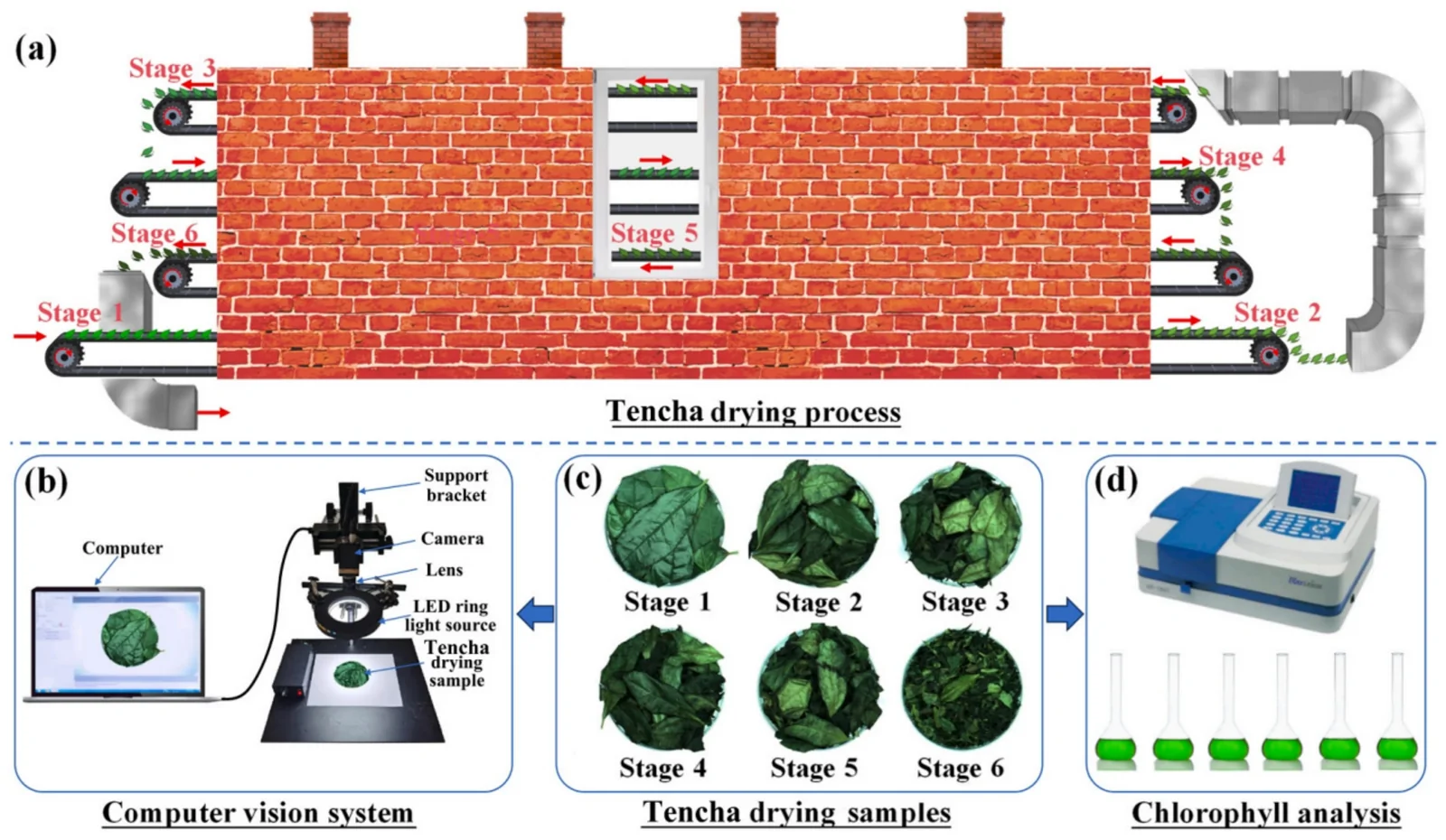
Flowchart of image acquisition and chlorophyll content measurement for tencha drying samples [157]. (**a**)—Tencha drying process; (**b**)—Image acquisition system; (**c**)—Images of tencha drying samples at six different drying stages; (**d**)—Chlorophyll content measurement by spectrophotometer.

Process monitoring and online quality inspection are now frequently carried out using multi-source sensing and intelligent recognition [155]. At present, some technologies for real-time measurement of the external quality, chemical components and hardness of agricultural products include machine vision, near-infrared spectroscopy, X-ray inspection, microwave sensing and tactile sensors. In 2025, Li et al. [156] presented a dynamic corn kernel counting system that integrates YOLOv8 with ByteTrack. The former found kernel targets; the latter handled multi-target trajectory association and tracking. The system achieved high-precision grain counting in cluttered environments with high processing speed. In 2025, Li et al. [157] built an improved ResNet computer vision model. Images of vine tea drying samples were taken by a visual system, as shown in Figure 8. These images were combined with spectrophotometric data to establish a nonlinear regression model. The model enabled rapid and non-destructive prediction of chlorophyll content changes. In 2025, Sun et al. [158] employed portable near-infrared spectroscopy and the lightweight MobileNetV4 model. Traditional spectral pre-processing methods, such as Savitzky–Golay filtering, standard normal variate (SNV) transformation and band dimensionality reduction, were combined with depthwise separable convolutions and attention mechanisms. These preprocessing methods reduced measurement noise, while the deep model extracted informative spectral features for prediction. An end-to-end classification accuracy of 98.33% was obtained. In 2026, Kang et al. [159] applied microwave nondestructive testing to the detection of boric acid adulteration in wheat flour. By measuring the amplitude attenuation and phase response of microwave signals across the 2.5-11.5 GHz frequency range and processing the resulting spectra with a random forest classifier whose feature set and hyperparameters were optimized using the whale optimization algorithm (RF-WOA), the method achieved semi-quantitative grading detection at 94.6% accuracy. In 2026, to enable the grading of kiwifruit firmness, Cai et al. [160] developed a non-destructive testing system that integrates a flexible tactile sensor array with a bionic gripper. By collecting multidimensional temporal tactile data and combining time-convolutional networks, graph-convolutional networks, and hierarchical graph pooling methods, they achieved high-precision classification with an accuracy rate of 99.59%.

#### 3.7.2. Adaptive Control of Drying Processes

Regarding intelligent control of the drying process, it should be pointed out that drying is required to reduce the moisture content of agricultural products and improve their storage stability and suitability for deep-processing. Control of the drying process for grains and agricultural products involves complex heat transfer, moisture migration and quality changes, and is inherently non-linear, has significant time delays and strong coupling. Fixed-parameter control schemes and operator-dependent empirical adjustments are the standard approaches in much of the industry; they handle this complexity poorly when the moisture content of the incoming material varies, when the ambient temperature and humidity change, or when the physical state of the material (shrinkage, case hardening) affects the drying kinetics [161]. In 2025, Pei et al. [162] built a multi-sensor fusion control system for combined far-infrared and hot-air drying of ginger. An electronic nose and computer vision were integrated to achieve the online perception of flavor and color. By establishing a two-stage fuzzy control model through correlation analysis, the dynamic adjustment of heating power and the synergistic optimization of product quality were realized. In 2025, based on near-infrared spectroscopy and low-field nuclear magnetic resonance, Sun et al. [163] developed a non-destructive online monitoring approach for tracking color changes during carrot drying. A threshold-triggering mechanism and MPC were introduced to attain the adaptive optimization of microwave power and temperature. In 2026, by integrating machine vision and odor sensing information, Peng et al. [164] employed a control system for microwave and hot-air drying. A PSO-optimized Least Squares SVM was utilized to establish an inverse model, which was integrated with PID feedback control to achieve two-stage dynamic temperature regulation and online optimization. The retention of active ingredients and the overall drying quality were improved. In 2026, Song et al. [165] used a Gaussian Process Regression (GPR)-MPC framework. A mechanistic model was used in conjunction with data-driven methods to perform moisture-content prediction and error compensation for adaptive adjustment and precise control of drying process parameters.

#### 3.7.3. Data-Driven Performance Prediction and Process Optimization

ML methods can predict process performance, optimize energy consumption, and regulate product quality in intelligent drying and processing systems [166]. Artificial Neural Networks (ANNs) can form a nonlinear mapping function between input parameters and output responses. Therefore, they are very suitable for multi-objective prediction problems, such as drying time, color change, nutritional retention and effective diffusion coefficients. In 2025, El-Mesery et al. [167] built a prediction-optimization model for microwave-hot-air coupled drying by combining ANN and self-organizing maps (SOM). The above model was used to investigate the effects of temperature, air velocity and power on important quality indicators of okra, such as color change, water activity and vitamin C content. Multi-parameter synergistic optimization was achieved via PCA. In 2026, Han et al. [168] built a combined vacuum freeze-drying and hot-air drying system. Low-field nuclear magnetic resonance (LF-NMR) was used to monitor moisture migration, and multi-source detection technologies, such as electronic nose and headspace gas chromatography–ion mobility spectrometry (HS-GC-IMS), were combined with RF and odor activity values to identify key volatile compounds. The regulatory mechanisms of different moisture turning points on aroma components and metabolic pathways were revealed. In 2026, El-Mesery et al. [169,170] investigated infrared and microwave drying systems. By integrating thermodynamic analysis, thin-layer drying models, and Fick’s diffusion theory with ANN, SOM, and PCA, predictive models for drying time, energy consumption, and effective diffusion coefficients were constructed. This approach enabled the precise characterization of heat and mass transfer processes as well as the synergistic optimization of multiple parameters.

As shown in Table 11, the integration of machine vision, portable near-infrared spectroscopy, microwave sensing, and tactile sensor arrays with advanced control strategies (MPC, fuzzy logic, GPR-MPC) and machine learning-driven quality prediction models (ANN-SOM, PSO-LSSVM, MobileNetV4) constitutes the dominant paradigm for achieving quality-driven processing.

## 4. Challenges and Prospects of Autonomous Agricultural Systems

### 4.1. Bottlenecks in Cross-Domain Generalization and Perception Robustness Under Complex Agricultural Environments

The single factor that most consistently limits the practical application of AASs is the complexity and variability of the agricultural environment itself. Perception technologies, including machine vision, LiDAR, and RGB-D cameras, have been applied with considerable success to crop identification, guidance line extraction, canopy analysis, and quality monitoring [171]. The difficulty is that accuracy achieved under one set of conditions does not reliably transfer to another. When the geographic region, crop variety, growth stage, or operation type changes, the recognition accuracy of the perception model tends to fall while false detection and missed detection rates rise [172,173,174]. The two reasons are as follows. Firstly, agricultural environments are inherently non-stationary; that is, their statistical attributes vary across space, time and under different crop conditions in an unpredictable manner [175]. Secondly, a single sensor modality provides incomplete observations, and the missing information may be critical for maintaining system performance under varying conditions.

Future research should focus on the transition from single-modality perception to multi-source heterogeneous fusion. On the one hand, large-scale and diverse field datasets covering multiple regions, soil conditions, climates, crop types, and operating modes should be established to evaluate performance variability, while domain adaptation and transfer learning techniques should be employed to improve model generalization in real-world environments. Meanwhile, mechanisms for multi-source information fusion should be further developed. Multi-modal data, such as visual, LiDAR, spectral and IMU data, as well as machine operating state information, should be closely coupled through spatiotemporal alignment and cross-modal feature fusion modeling. Such efforts will facilitate the development of high-stability, all-weather perception systems that provide reliable environmental awareness and decision support under challenging agricultural conditions.

### 4.2. Constraint Mechanisms for Explainability and Trustworthiness of Data-Driven Intelligent Decision-Making

Intelligent decision-making serves as the core element in facilitating the evolution of AASs from automated execution to autonomous operation. With the advancement of ML and DL, data-driven approaches have been extensively applied in fields such as irrigation scheduling, variable-rate fertilization, and harvest status prediction, demonstrating high predictive accuracy in specific tasks [176,177]. Unlike the control layer, which focuses on the modeling of physical processes, the challenge of intelligent decision-making is essentially a matter of knowledge representation and reasoning. However, mainstream data-driven methods rely on black-box models that lack interpretability regarding the decision-making process [178,179]. The inherent connections between decision outcomes and factors such as crop physiological processes, environmental variables, and resource utilization are difficult to reveal, which restricts their trustworthy application in agricultural production [180].

The future development of intelligent decision-making systems should shift from purely data-driven fitting to a knowledge-data collaborative approach, emphasizing the construction of hybrid intelligence models that integrate agronomic mechanistic knowledge with data-driven techniques. CGM, agronomic expertise, and management rules serve as the fundamental prior knowledge base for agricultural systems. Crop growth mechanisms, soil–water–nutrient cycling processes, and agronomic management strategies can be embedded into data-driven models. Thereby, mechanistic constraints are imposed on decision variables. Prediction accuracy is improved while model interpretability and generalization capability are enhanced. Furthermore, the introduction of explainable artificial intelligence methods allows for multidimensional analysis of model operations and outputs. Decision outcomes can be quantitatively interpreted through key indicators such as crop water requirements, nutrient status, and energy consumption, providing transparent decision-making evidence for agricultural managers.

### 4.3. Challenges in Adaptation and Stability for Precise Control Under Multi-Disturbance Coupled Conditions

The operational processes of AASs are characterized by unstructured environments, intense disturbances, and time-varying dynamics. Key operational parameters such as tillage depth, seeding depth, and spray rate are susceptible to the coupled effects of multiple factors, including soil resistance, implement attitude, feed rate, and crop moisture content [181,182,183]. Consequently, the system dynamic characteristics become highly complex and difficult to model accurately. Although traditional methods are well-established in engineering applications, these approaches are often dependent on empirical tuning, and their global adaptability to complex dynamic environments is found to be limited [184]. Advanced control methods, including MPC, neural network control, RL, and LADRC, theoretically offer superior predictive capability and disturbance rejection performance. However, their practical application remains dependent on high-precision system models or large-scale training data.

The future development of precision control technology should transition from traditional empirical tuning toward physics-informed and mechanism-constrained control frameworks. On the one hand, mechanism-constrained control models tailored for multi-physics field coupling processes should be constructed. Through the incorporation of physical models describing soil mechanics, crop fluid dynamics, and implement dynamics, a physical account of key system state variables can be obtained. Data-driven methods should concurrently be combined with these models to compensate for components that are difficult to characterize mechanistically or that involve substantial uncertainty, thereby establishing a control framework that unifies mechanistic constraints with data-driven techniques. Furthermore, the introduction of digital twin technology allows for real-time prediction and dynamic optimization of the operational process through virtual-real mapping. The control system is capable of updating model parameters online in response to environmental changes, improving operational accuracy and environmental adaptability.

## Figures and Tables

**Table 1:** The advantages and disadvantages of multi-source perception technologies.

Technology	Theory	Advantage	Disadvantage
GNSS	Measures signal propagation time to derive pseudo-range observations; computes precise position, velocity, and time via multi-satellite constellation solving.	Broad coverage; enables all-weather localization; mature and widely deployed.	Susceptible to atmospheric error and multipath effects; performance degrades in occluded environments; limited single-point accuracy.
RGB-D camera	Integrates red, green, and blue images to generate 3D representations of crops using computer vision techniques.	High perceptual precision; supports 3D modeling and object identification.	Sensitive to illumination; high cost; stability decreases in complex environments.
LiDAR	Measures distances by calculating the time-of-flight of emitted laser pulses to generate 3D environmental point clouds.	High 3D sensing accuracy; suitable for complex terrains.	High cost; sensitive to environmental conditions; complex data processing.
Multispectral/ hyperspectral sensor	Captures spectral information across multiple bands to invert the physiological status of crops and soil.	Non-contact and non-destructive; suitable for large-scale monitoring with rich information dimensionality.	Complex data processing; high calibration requirements; high equipment cost.
Ultrasonic sensor	Determines distances by calculating sound wave propagation time for obstacle detection.	Low cost; simple structure; suitable for short-range measurements.	Susceptible to noise interference; material-sensitive; limited ranging coverage.
IMU	Estimates pose and motion states by measuring linear acceleration and angular velocity via accelerometers and gyroscopes.	Rapid response; independent of external environment; ideal for dynamic pose estimation.	Subject to cumulative errors; necessitates fusion with GNSS.
Soil sensor	Measures soil moisture, electrical conductivity, and temperature through electrical or physical properties.	Enables direct acquisition of in situ soil information.	Limited spatial coverage; requires sensor network deployment; sensitive to environmental interference.
Spectral analysis	Performs quantitative analysis based on the reflection/absorption characteristics of substances across the electromagnetic spectrum.	Non-destructive and rapid; ideal for batch inspection; highly compatible with ML.	High data dimensionality; complex preprocessing; high equipment cost.
Traditional image processing	Achieves object recognition via segmentation, thresholding, and feature extraction.	Simplified methodology; high interpretability; low computational requirements.	Poor adaptability to complex environments; limited generalization capability.
DL	Automatically learns hierarchical features via neural networks for classification and regression tasks.	Strong robustness; high accuracy; well-suited for complex scenarios.	Heavy reliance on large-scale annotated datasets; limited model interpretability.

**Table 2:** Comparison of multi-source sensor fusion strategies.

Fusion Strategy	Typical Methods	Advantages	Disadvantage
Data-level fusion	Raw data integration, weighted fusion after calibration and registration.	Preserves raw information and minimizes information loss.	Requires accurate alignment and high computational resources.
Feature-level fusion	CNN, Transformer, attention mechanism.	Captures nonlinear correlations among heterogeneous sensor features; widely used in deep learning-based perception.	Requires large-scale datasets and computational resources.
Decision-level fusion	Bayesian inference, D-S theory, weighted voting.	High robustness against sensor uncertainty and individual sensor failure.	May lose low-level information during independent decision generation.

**Table 3:** The advantages and disadvantages of intelligent decision-making technologies.

Technology	Theory	Advantage	Disadvantage
Multivariate statistical analysis	Performs dimensionality reduction and correlation modeling based on statistical methods to reveal intrinsic relationships and feature extraction.	Clear model structure; strong interpretability; suitable for variable screening and dimensionality reduction; provides a data foundation for ML.	Difficult to characterize complex nonlinear relationships; sensitive to data quality and variable selection.
Operational research	Solves for optimal decision schemes by constructing objective functions and constraints based on mathematical optimization models.	Suitable for resource allocation and path planning; supports multi-objective optimization; mature theoretical framework.	Complex modeling; highly dependent on objective and constraint settings; intensive computational requirements for complex problems.
Expert system	Codifies expert experience into rules based on knowledge engineering and implements decision-making through inference mechanisms.	Clear logic; strong interpretability; suitable for scenarios with well-defined rules.	High maintenance cost for knowledge base construction; insufficient adaptability to complex, dynamic environments.
CGM	Constructs coupled climate-soil-management models based on physiological and ecological processes to simulate crop growth and yield formation.	Clear physical mechanisms; suitable for long-term prediction and scenario analysis; supports management optimization.	Large number of parameters and complex calibration; limited regional adaptability and real-time performance.
Spatial decision technology	Acquires and analyzes spatial data via remote sensing and geographic information system to enable inversion of crop/soil information and spatial decision-making.	Wide coverage; strong spatial representation capability; supports dynamic monitoring and prescription map generation.	Susceptible to weather and resolution limitations; high inversion errors and ground verification costs.
ML	Learns input-output mapping relationships based on data-driven approaches to achieve prediction and pattern recognition.	Suitable for nonlinear problems; high prediction accuracy; supports multi-source data fusion.	Heavy reliance on high-quality data; insufficient interpretability; limited generalization capability.
RL	Learns optimal decision strategies through interaction based on a “state-action-reward” mechanism.	Suitable for dynamic and sequential decision-making; possesses self-adaptive optimization capabilities.	High training costs; significant trial-and-error risks; stability and safety require further improvement.

**Table 4:** The advantages and disadvantages of precision control technologies.

Technology	Theory	Advantage	Disadvantage
PID control	Regulates system error based on proportional, integral, and derivative terms to achieve closed-loop control.	Simple structure; ease of implementation; good stability; mature engineering application.	Parameter tuning is reliant on experience; insufficient adaptability to highly nonlinear and time-varying systems.
Fuzzy control	Implements control strategies without the need for precise models based on fuzzy rules and expert experience.	Independent of precise models; high adaptability; suitable for nonlinear systems.	Rule design is dependent on experience; limited control precision; difficulty in achieving global optimality.
Linear quadratic regulator	Achieves optimal state feedback control by constructing state-space models and performance index functions.	Superior control performance; well-established theoretical framework; suitable for optimized control of linear systems.	Dependence on precise mathematical models; limited adaptability to nonlinear and uncertain systems.
SMC	Forces system states to converge to and remain near a sliding mode surface by designing switching control laws.	High robustness; strong anti-disturbance capability; suitable for uncertain systems.	Susceptible to chattering phenomena, which affects the smoothness and service life of actuators.
Linear active disturbance rejection control (LADRC)	Estimates and compensates for total system disturbances online using an extended state observer to achieve disturbance decoupling.	Low model dependency; strong anti-disturbance capability; suitable for uncertain and time-varying systems.	Complex parameter design; relatively novel theoretical framework; lack of unified standards for engineering application.
MPC	Executes rolling prediction and optimization of future behavior based on system models to achieve multi-variable constrained control.	Capable of handling multi-variable coupling and constraints; strong predictive capability; high control precision.	High computational complexity; demanding requirements for model accuracy and computational resources.
Neural network control	Performs system modeling and control decision-making based on data-driven neural network models.	Capable of approximating complex nonlinear systems; possesses self-learning and adaptive capabilities.	Reliance on large amounts of training data; weak interpretability; difficulty in stability and convergence analysis.

**Table 5:** Representative studies on multi-sensor fusion-based perception, adaptive depth control, and soil–tool interaction modeling for autonomous tillage systems.

Ref.	Method	Research Problem	Performance Indicators
[59]	Fuzzy logic control; engine load characteristic model; cooperative depth-load regulation	Engine overload during deep tillage due to soil resistance variation	Bench test under increasing soil resistance; tillage depth reduced by 26% (250 → 185 mm) from the set 250 mm target
[60]	Fuzzy-PID control; angular displacement and resistance sensing; electrohydraulic actuation	Inadequate depth consistency and soil fragmentation in electric tillage	Field test (greenhouse rotary tillage); tillage-depth stability variation reduced by 24% vs. conventional PID (depth SD 6.7 vs. 8.8 mm)
[61]	EKF; online model parameter calibration; GNSS; PID control	Vibration-induced measurement errors in dynamic tillage depth estimation	Field test (Shangshi Farm, rice stubble); tillage depth controlled with RMS error < 1 cm (0.847/0.365 cm)
[62]	LADRC with extended state observer; attitude and grating sensor fusion; online disturbance estimation and cancelation	Poor disturbance rejection and control accuracy under terrain-induced perturbations	Field test (tea garden, 0.5/0.8 km/h, 80/100 mm); tillage-depth rate-of-change reduced by 68.9% vs. fuzzy PID (SD 3.2 vs. 10.5 mm)
[64]	Tension sensing; adaptive threshold control; automatic vibration mode switching (self-excited/forced)	High operating resistance and depth instability in subsoiling operations	Field test (black soil, 35–45 cm, 2–4 km/h); traction resistance reduced by 9.6–18.9% vs. conventional subsoiling
[66]	DEM; Hertz-Mindlin contact model; parallel bond model; soil bin test calibration	Difficulty in predicting mechanical response of complex subsoiling tools	Indoor soil-bin validation (sandy clay loam, 0.25 m/s, 150 mm); DEM draft-force relative error −2.0%
[67]	DEM coupled with multi-body dynamics; full-scale tractor-plow-soil model; layered soil and tire calibration	Difficulty in accurate draft force prediction under varying tillage depths	Field test (rice paddy, moldboard plow); draft-force prediction accuracy 90.8%
[68]	EEPA contact model; DEM simulation; multi-soil-type calibration	Limited applicability of draft force models across different soil textures	Field test; DEM draft-force prediction accuracy 86.75% (loam) and 74.51% (clay loam)

**Table 6:** Representative studies on vision-based autonomous navigation, advanced path tracking control, and multi-sensor precision seed placement for intelligent planting systems.

Ref.	Method	Research Problem	Performance Indicators
[73]	Lightweight CNN; parameter-free attention mechanism; improved feature extraction; RANSAC line fitting	Reduced navigation accuracy under seedling deficiency conditions in ridge-planted vegetables	Broccoli seedling-row detection; SN-CNN mAP@0.5 = 94.6%
[76]	YOLOv8 and ByteTrack multi-object tracking; data augmentation; edge deployment optimization	Robust real-time trunk detection in complex orchard environments with varying illumination	Orchard (apple) trunk detection; Trunk-Seek mAP@0.5 = 98.9%
[78]	Adaptive SMC; RBF neural network uncertainty compensation	Path tracking degradation due to field sideslip and uncertain disturbances	Paddy field test; average lateral deviation 3.2 cm vs. 9.1 cm (traditional SMC)
[81]	Clipping sliding window filter for ultrasonic outlier suppression; fuzzy look-ahead distance decision; pure pursuit controller	Ranging errors and path tracking instability on rough ridged soil surfaces	Greenhouse field test (strawberry ridges); mean absolute lateral deviation 10.56 mm at 62.5 mm/s vs. 13.11 mm (UPP)
[84]	MPC; sideslip angle integrated into kinematic model; Carsim-Simulink co-simulation	Lateral tracking error and sideslip oscillation in agricultural vehicle path tracking	CarSim–Simulink co-simulation; lateral-error mean reduced by 58.7% (U-path) vs. conventional MPC
[86]	Multi-sensor fusion; Finite state machine logic; coordinated transverse-longitudinal motion control	Low seedling placement success rate due to poor tray positioning and pick-drop coordination	72-hole-tray transplanting; average seedling extraction success rate 95%
[88]	Laser and array sensor fusion; fuzzy PID control with online parameter adaptation	Seeding depth inconsistency under complex terrain variations in wheat planting	Bench test (wheat row planter, 40 mm target); sowing-depth error reduced by 10.7–22.9% vs. no control
[89]	Digital twin; virtual prototyping; MSC Adams View 2020 and MATLAB Simulink R2024a co-simulation; feedback control and parameter optimization	Difficulty in precise trajectory tracking and position control of seedling transmission	Digital twin (Adams/MATLAB vs. physical prototype); maximum linear positioning discrepancy 0.95 mm
[90]	Ultrasonic ranging; echo characteristic analysis; error correction algorithm	Transplanting depth fluctuation caused by rough ridged soil surface variation	Field trial (Zhenjiang, 1 km/h); planting-depth qualification rate 94.4% (average 79.86 vs. 80 mm target)

**Table 7:** Representative studies on data-driven water demand prediction, sensor-based closed-loop regulation, and hydraulic performance optimization for precision irrigation systems.

Ref.	Method	Research Problem	Performance Indicators
[104]	GEP; 17 meteorological factor combinations; multi-climate validation	Limited meteorological inputs for ET estimation	Monthly ET_0_ prediction (Skardu, humid climate); GEP-13 testing R^2^ = 0.99, RMSE = 0.27 mm/month
[105]	MRMR feature selection; neural network models	Nonlinearity and limited data in long-term ET prediction	Long-term crop water-demand prediction; Wi-ANN testing R^2^ = 0.977, RMSE = 6.423 mm
[106]	DSSAT framework (CERES and CROPGRO modules); ML coupling; meteorological, soil, and water supply constraint integration	Temporal constraints of canal water supply limiting irrigation scheduling optimization	Dryland cotton (Xinjiang, canal constraints); cotton yield increased by 8.5% (to 9724 kg/ha) vs. traditional irrigation
[107]	RF; SVR; MLP; multi-model ensemble	Time-consuming experimental measurement of sprinkler performance parameters	Sprinkler uniformity prediction; XGBoost CU R^2^ = 0.88, RMSE = 1.79%
[108]	ZigBee sensor network; dynamic ET model; threshold-based control	Delayed irrigation decisions in soilless cultivation	Lettuce soilless culture; fresh weight increased by ≥16.60% (spring) vs. manual control
[109]	Genetic algorithm optimization; variable-frequency pump control	Uneven pressure distribution and high energy consumption	Sprinkler scheduling (GA + VFD); irrigation energy cost reduced by ~18% vs. optimal branch-pipe scheduling
[111]	Rotating sprinkler design; flow breakup optimization	Low sprinkler uniformity	Novel rotating sprinkler; coefficient of uniformity improved by 54.89% (device B @ 100 kPa vs. original)
[112]	Water application model; multi-parameter optimization	Poor uniformity caused by parameter mismatch	Linear-move irrigation; maximum combined uniformity coefficient = 95.0% (16.0 × 6.0 mm nozzle, 180°, 1.6R spacing)

**Table 8:** Representative studies on spectral-perception-based crop nutrient sensing, model-driven variable-rate decision-making, and adaptive fertilization control for precision nutrient management systems.

Ref.	Method	Research Problem	Performance Indicators
[117]	Multi-dimensional UAV feature fusion (multispectral and RGB); grey relational analysis for variable screening; parallel multi-branch DNN (DNN-F2)	Difficulty in high-precision rice nitrogen status inversion from UAV imagery	UAV multispectral LNC estimation (rice); RIDGE-SPA R^2^ = 0.76, RMSE = 10.33%
[118]	Multi-source hyperspectral spectral indices; gradient boosting regression model	Simultaneous estimation of chlorophyll, yield, and quality under varying nitrogen regimes	Sugar beet chlorophyll prediction; GBR test R^2^ = 0.65 (RMSE = 0.354 mg/g FW)
[119]	Isolation Forest; Local spatial autocorrelation; hybrid global-local anomaly detection framework	Insufficient accuracy in identifying outliers in large-scale soil nitrogen datasets	Soil total-nitrogen anomaly detection (25,930 observations); correct detection rate 99.97%
[120]	UAV multispectral imagery; SVM; RF; multi-condition validation	Limited generalization of chlorophyll estimation models across crop varieties and growth stages	UAV multispectral LCC estimation (winter wheat); VI–LCC correlation r = 0.95 (VARI/VEG/NDVI)
[121]	Improved lightweight YOLOv5s-seg; instance segmentation; time-series imaging; flow rate calculation	Difficulty in real-time particle flow rate measurement for centrifugal variable-rate spreaders	Fertilizer discharge flow-rate detection; improved YOLOv5s-seg mAP = 95.9% (6.66 MB)
[124]	Piecewise linear interpolation; dynamic motor-drive-to-shaft-speed mapping; self-calibration; multi-parameter collaborative control	Inaccurate fertilizer rate control due to nonlinear discharge characteristics	Self-calibration (vs. linear model); steady-state shaft-speed error 0.13 vs. 0.36 r/min
[125]	DSSAT crop model; XGBoost algorithm; ecological stoichiometry theory constraints; cooperative NP ratio optimization	Suboptimal nitrogen-phosphorus ratios limiting nutrient use efficiency	Optimal N-P treatment (90% N + 80% P); cotton yield increased by 8.3% vs. local recommendation
[126]	DSSAT model; Genetic algorithm; staged nitrogen optimization strategy	Excessive nitrogen application with marginal yield returns in cotton production	DSSAT + genetic algorithm; lint cotton yield increased by 10.9% vs. highest field group

**Table 9:** Representative studies on deep learning-based target perception, disturbance-robust spray regulation, and intelligent precision application for autonomous crop protection systems.

Ref.	Method	Research Problem	Performance Indicators
[129]	Spectral reflectance and fluorescence characteristics; optimized multi-band light source and detection parameters; low-cost sensor design	High cost and complexity of vegetation detection sensors for precision spraying	Low-cost fluorescence sensor; vegetation detection accuracy 100% (dark and outdoor)
[130]	Automatic threshold segmentation; color feature extraction; multi-growth-stage validation	Difficulty in differentiating cotton seedlings from weeds at various growth stages	Cotton-field weed identification; overall recognition rate 82.1% (color features + OTSU)
[131]	LiDAR point cloud processing; clustering segmentation; geometric reconstruction; coupled multifactor regression	Challenging high-precision inversion of orchard canopy structural parameters	Orchard canopy LiDAR; leaf-area-density model R^2^ = 0.96 (mean relative error 11.58%, lab)
[132]	Refined YOLOv5 detection; visual servoing; robotic arm docking; automatic liquid transfer	Limited endurance and efficiency of unmanned field sprayers due to manual refill requirements	Liquid pesticide-replenishment docking; average docking time 35.64 s
[134]	CNN real-time weed identification; closed-loop control integrating image acquisition with spray actuation hardware	Excessive agrochemical consumption and off-target deposition caused by conventional uniform spraying	Targeted weed spraying (strawberry); VGG-16 validation accuracy 0.97
[135]	LADRC; Extended state observer; PSO for parameter tuning	Time-varying disturbances and model uncertainty in spray rate regulation	Field spray control (12 m boom, 6/8/10 km/h); LADRC spray error −2.6% to 3.7% vs. PID −7.8% to 9.2%
[136]	Ultrasonic sensing array; data-driven signal processing; dynamic boom height detection and regulation	Boom height instability causing uneven spray deposition in field conditions	Field boom sprayer; maximum boom-height variation exceeded 50 cm
[137]	Real-time kinematic (RTK)-GNSS augmentation; wheel odometry positioning; lag compensation model; PID flow closed-loop control	Overlaps and omissions in spray coverage during autonomous field spraying	GNSS-guided breakpoint spraying; RTK + odometer breakpoint accuracy 9.29 cm vs. 25.65 cm (RTK-only)

**Table 10:** Representative studies on multi-modal sensor fusion, machine learning-based fault diagnosis, and model-based adaptive process control for intelligent harvesting systems.

Ref.	Method	Research Problem	Performance Indicators
[140]	Improved YOLOX network; RGB-D image fusion strategy; 3D localization	Low target recognition accuracy and real-time performance in complex orchard environments	Apple detection (RGB-D); improved YOLOX mAP = 94.09%
[141]	RGB-D camera; YOLOv5 instance segmentation; Jetson Xavier NX edge computing; grain pile height mapping	Manual grain unloading operation reducing harvesting efficiency and causing grain spillage	Rice combine grain unloading (stereo vision); unloading success rate 90%
[142]	Binocular vision; improved YOLOv5s; C3Res2NetBlock multi-scale feature extraction; P2 small-target detection layer; efficient multi-scale attention	Difficulty in simultaneous wheat ear detection, stubble height estimation, and feed rate prediction	Wheat feed-rate prediction; improved YOLOv5s mAP50 = 78.1% (+6.8% vs. baseline)
[143]	Lightweight semantic segmentation (StarNet-RiceSeg); Star Operation; residual spatial attention; TensorRT deployment on Jetson Xavier NX	Real-time accurate identification of rice lodging areas for harvesting path planning	Rice lodging detection; StarNet-RiceSeg mIoU = 94.42%
[144]	ImpurityWeightNet; multimodal fusion of image features + structural attributes (area, aspect ratio, perimeter)	Difficulty in high-precision online estimation of impurity mass in combine harvester grain flow	Rice impurity-mass estimation; ImpurityWeightNet R^2^ = 0.8644 vs. vision-only R^2^ = 0.3524
[145]	Multi-point vibration signal fusion; time-frequency domain feature extraction; PCA + ELM/RF/SVM classifiers	Delayed detection of threshing overload and clogging in combine harvesters	Threshing load recognition; RF-KPCA prediction accuracy 98%
[149]	Multi-component slip rate monitoring; IPSO-optimized SVM fault prediction; fuzzy logic speed adaptation	Fault-induced downtime and suboptimal speed regulation in unmanned harvesting	Unmanned combine fault prediction; IPSO-SVM accuracy 98.59% (+8.44% vs. PSO-SVM)
[150]	Permanent magnet synchronous motor electric drive; RLS inertia identification; PI parameter self-tuning	Threshing drum speed instability under variable crop feed rates	Electrically driven threshing cylinder; startup overshoot reduced by 11.1% vs. fixed-parameter PI
[151]	Hydraulic variable-diameter drum; real-time oil pressure and clearance monitoring; adaptive variable-universe fuzzy PID	Fixed concave clearance unable to adapt to varying feed rates and crop conditions	Hydraulic variable-diameter threshing drum; entrainment loss 0.16% (field verification)
[152]	RTK-GPS positioning; feed-forward PID steering control; look-ahead Ackermann path-tracking algorithm	Path tracking difficulty for large, rear-wheel-steered combine harvesters	Large rear-wheel-steered combine; average lateral deviation 4.3 cm (straight AB-line)

**Table 11:** Representative studies on multi-sensor quality inspection, intelligent process optimization, and adaptive control for autonomous post-harvest processing systems.

Ref.	Method	Research Problem	Performance Indicators
[156]	YOLOv8 detection; ByteTrack multi-target trajectory association and tracking; dynamic counting	Inaccurate grain counting under complex occlusion and high-speed motion	Maize kernel counting; tracking/counting accuracy > 99% (99.3–100% across trials)
[157]	Improved ResNet computer vision model; spectrophotometric data integration; nonlinear regression	Difficulty in rapid non-destructive chlorophyll content prediction during tea drying	Tencha chlorophyll prediction (drying); DCAM-ResNet Rp = 0.9656 (Chl-a)
[158]	Portable NIR spectroscopy; MobileNetV4 lightweight model; Savitzky–Golay filtering; depthwise separable convolutions	Need for rapid non-destructive identification of coffee processing methods	Coffee-bean processing-method identification; MobileNetV4 accuracy 98.33%
[159]	Microwave nondestructive testing (2.5–11.5 GHz); amplitude attenuation and phase response measurement; RF-WOA	Difficulty in rapid semi-quantitative detection of adulterants in wheat flour	Wheat-flour borax adulteration detection; RF-WOA classification accuracy 94.6%
[160]	Flexible tactile sensor array; bionic gripper; temporal convolutional network; graph convolutional network; hierarchical graph pooling	Non-destructive firmness evaluation challenging for fruit quality grading	Kiwifruit firmness grading; GCN accuracy 99.59% (3 ripeness stages)
[162]	Electronic nose + computer vision; online flavor and color perception; two-stage fuzzy control; correlation analysis	Coupled nonlinear drying dynamics and quality degradation during ginger processing	Ginger far-infrared hot-air drying; FLC drying time reduced by 40.6% vs. 50 °C constant temperature
[163]	NIR spectroscopy and LF-NMR; online color change monitoring; threshold triggering; MPC	Color degradation and quality loss during carrot drying under fixed control parameters	Carrot drying color control; BP-ANN browning calibration R^2^ = 0.9220
[164]	Machine vision + electronic nose sensing; PSO-optimized Least Squares SVM inverse model; PID feedback; two-stage dynamic temperature regulation	Difficult tradeoff between drying speed and active ingredient retention in medicinal plant drying	Gastrodia elata two-stage drying; overall quality score improved by 15.13% vs. M70-H30 °C

## Data Availability

No new data were created or analyzed in this study. Data sharing is not applicable to this article.
